# *RTL4*, a Retrovirus-Derived Gene Implicated in Autism Spectrum Disorder, Is a Microglial Gene That Responds to Noradrenaline in the Postnatal Brain

**DOI:** 10.3390/ijms252413738

**Published:** 2024-12-23

**Authors:** Fumitoshi Ishino, Johbu Itoh, Ayumi Matsuzawa, Masahito Irie, Toru Suzuki, Yuichi Hiraoka, Masanobu Yoshikawa, Tomoko Kaneko-Ishino

**Affiliations:** 1 Department of Epigenetics, Medical Research Institute (MRI), Tokyo Medical and Dental University (TMDU), Tokyo 113-8510, Japan; am@logomixgenomics.com (A.M.); irie@dna-gib.com (M.I.); 2 Department of Neurology, School of Medicine, Tokai University School of Medicine, Isehara 259-1193, Japan; itohj@tokai.ac.jp; 3 Faculty of Nursing, Tokai University School of Medicine, Isehara 259-1193, Japan; 4 Laboratory of Genome Editing for Biomedical Research, Medical Research Institute (MRI), Tokyo Medical and Dental University (TMDU), Tokyo 113-8510, Japan; t-suzuki.lra@mri.tmd.ac.jp (T.S.); hiraoka@m.u-tokyo.ac.jp (Y.H.); 5 Laboratory of Molecular Neuroscience, Medical Research Institute (MRI), Tokyo Medical and Dental University (TMDU), Tokyo 113-8510, Japan; 6 Department of Clinical Pharmacology, Tokai University School of Medicine, Isehara 259-1193, Japan; yoshikaw@tokai.ac.jp

**Keywords:** retrovirus-derived genes, *Rtl4*/*Sirh11*, autism spectrum disorder (ASD), postanal brain, noradrenaline (NA), *Rtl4-Venus* knock-in mouse, microglia, exaptation, eutherian brain evolution

## Abstract

Retrotransposon Gag-like 4 (*RTL4*), a gene acquired from a retrovirus, is a causative gene in autism spectrum disorder. Its knockout mice exhibit increased impulsivity, impaired short-term spatial memory, failure to adapt to novel environments, and delayed noradrenaline (NA) recovery in the frontal cortex. However, due to its very low expression in the brain, it remains unknown which brain cells express RTL4 and its dynamics in relation to NA. We addressed these issues using knock-in mice carrying endogenous *Rtl4* fused to *Venus*, which encodes a fluorescent protein. The RTL4-Venus fusion protein was detected as a secreted protein in the midbrain, hypothalamus, hippocampus and amygdala in the postnatal brain. Its signal intensity was high during critical periods of neonatal adaptation to novel environments. It was upregulated by various stimuli, including isoproterenol administration, whereas it was decreased by anesthesia but was maintained by milnacipran administration, suggesting its highly sensitive response to stressors, possible dependence on the arousal state and involvement in the NA reuptake process. In vitro mixed glial culture experiments demonstrated that *Rtl4* is a microglial gene and suggested that RTL4 secretion responds rapidly to isoproterenol. Microglial RTL4 plays an important role in the NA response and possibly in the development of the NAergic neuronal network in the brain.

## 1. Introduction

Retrotransposon Gag-like (RTL)/sushi-ichi retrotransposon homolog (SIRH) genes are eutherian-specific genes, with the exception of therian-specific paternally expressed 10 (*PEG10*). They were acquired through three independent domestication events of a certain retrovirus-like retrotransposon (*Metaviridae*) during mammalian evolution [1,2,3,4,5,6,7,8,9,10,11]. It is reasonable to assume that they were originally derived from the group-specific antigen (GAG) and polymerase (POL) genes of a certain extinct retrovirus with a high degree of homology to the sushi-ichi retrotransposon, since the gypsy retrotransposon, which includes the sushi-ichi retrotransposon, is an infectious retrovirus in *Drosophila melanogaster* [12,13]. Of these eleven genes, at least ten RTL/SIRH genes are known to have specific essential and/or important functions in current eutherian developmental systems, such as the placenta and brain [8,10]. Among them, *RTL4* (also known as *SIRH11* or zinc finger CCHC domain-containing protein 16 (*ZCCHC16*)) has been implicated as a causative gene in autism spectrum disorder (ASD) through extensive screening of patients [14]. Lim et al. identified a family with a rare nonsense mutation in the X-linked gene *RTL4* (*ZCCHC16*) leading to ASD in a male proband [14]. There are two other examples of autistic boys with *ZCCHC16* mutations, one with a frameshift mutation and the other with a nonsense mutation (personal communication) [15,16]. Consistent with this, we previously demonstrated that *Rtl4* (*Sirh11/Zcchc16*) knockout (KO) mice exhibit increased impulsivity, decreased attention, poor working memory and reduced adaptation to novel environments [17]. They displayed low noradrenaline (NA) recovery in the frontal cortex, suggesting that the behavioral defects of the *Rtl4* KO mice are somehow related to a dysregulation of the NA system in the brain [17]. Despite its important role in the brain, several critical questions, such as which brain cells express the RTL4 protein and when, where, and how it functions in relation to NA, have long remained unelucidated, not least because *RTL4* expression is quite low throughout the body, including the brain [18,19].

NA-expressing neurons have been reported to play important roles in attention, vigilance, behavioral flexibility and modulation of cognition [20,21,22,23], and their activation occurs together with the cognitive shifts that facilitate the dynamic reorganization of target neural networks, allowing rapid behavioral adaptation to changing environmental demands [22]. NA is also the most well-documented neurotransmitter in stress experiments. NA has been reported to increase in the brain in response to various types of stressors [24], and the administration of the β-adrenergic receptor (AR) agonist isoproterenol significantly increases interleukin-1β in the brain [25,26] and cultured microglia [24,27]. Most of the secreted NA is reabsorbed by a noradrenaline transporter (NAT, also known as norepinephrine transporter, NET) located along the plasma membrane of the presynaptic neuron after it has completed its function of transmitting a neural impulse [28]. Therefore, the NAT-mediated reuptake of NA is critical for the adequate maintenance of intracellular NA stores in NA neurons [28]. Milnacipran is a serotonin (5-hydroxytryptamine, 5-HT)–NA reuptake inhibitor (SNRI) that is thought to increase the levels of synaptic 5-HT and NA by inhibiting their reuptake into neuronal cells, thereby exhibiting antidepressant and anxiolytic effects [29].

Neurotransmitters also play an important role in nervous system development, including the shaping and wiring of the nervous system during critical periods of development [30,31]. In particular, NA is postulated to be an important regulator of brain development, regulating the development of both NAergic neurons and their target areas during the early stages of development and affecting behaviors such as infant attachment leaning, aversion and fear learning, and synaptogenesis. Disruptions in this process can alter the trajectory of brain development, leading to long-term and even permanent changes in brain function and behavior later in life [31].

NA signals occur through three types of ARs, α1, α2 and β, each of which, in turn, has three subtypes, α1A, α1B and α1D; α2A, α2B and α2C; and β-1, β-2 and β-3, respectively. All of these are G protein-coupled receptors, with α1-ARs coupled to G_q_, which activate phospholipase C; α2-ARs coupled to G_i/o_, which inhibit adenylyl cyclase; and β-ARs coupled to G_s_, which stimulate adenylyl cyclase and have distinct pharmacological properties, molecular structures and signaling pathways [32]. These ARs are present on multiple cell types throughout the CNS, including astrocytes and microglia, as well as neurons [33,34]. The α1- and β-ARs are present at the postsynaptic site and generally mediate excitatory effects, whereas α2-ARs are present at both the pre- and postsynaptic sites and reduce NA release by decreasing neuronal excitability [35].

Microglia are the primary innate immune cells of the brain and play a central role in the immune response to various pathogens via a variety of Toll-like receptors (TLRs) [36,37] and/or in the clearance of various pathogen-associated molecular patterns (PAMPs), such as single-stranded (ss)RNA/double-stranded (ds)DNA (viruses), lipopolysaccharides (LPSs) (Gram-negative bacteria) and zymosan (fungi), by means of RTL/SIRH proteins. This is accomplished by the rapid capture- and degradation-associated activities of RTL5 (also known as SIRH8), RTL6 (also known as SIRH3) and RTL9 (also known as SIRH10), respectively [38,39]. Moreover, in the neonatal brain, microglia are involved in shaping neuronal circuits during development by regulating neurogenesis. They induce filopodia formation through direct contact with neurons and phagocytose supernumerary or unneeded synapses [36,40,41]. Therefore, they have been implicated as an important cause of several neurodevelopmental and neuropsychiatric disorders, including ASD [42,43,44,45].

In this work, using knock-in (KI) mice carrying a gene that fuses endogenous *Rtl4* with *Venus* (*Rtl4*CV mice) to produce the RTL4–Venus fusion protein at the C-terminus (RTL4CV), we investigated the dynamics of RTL4CV in the brain and its relationship to NA.

## 2. Results

### 2.1. RTL4 Is Expressed in the Postnatal Brain

**Figure 1:**
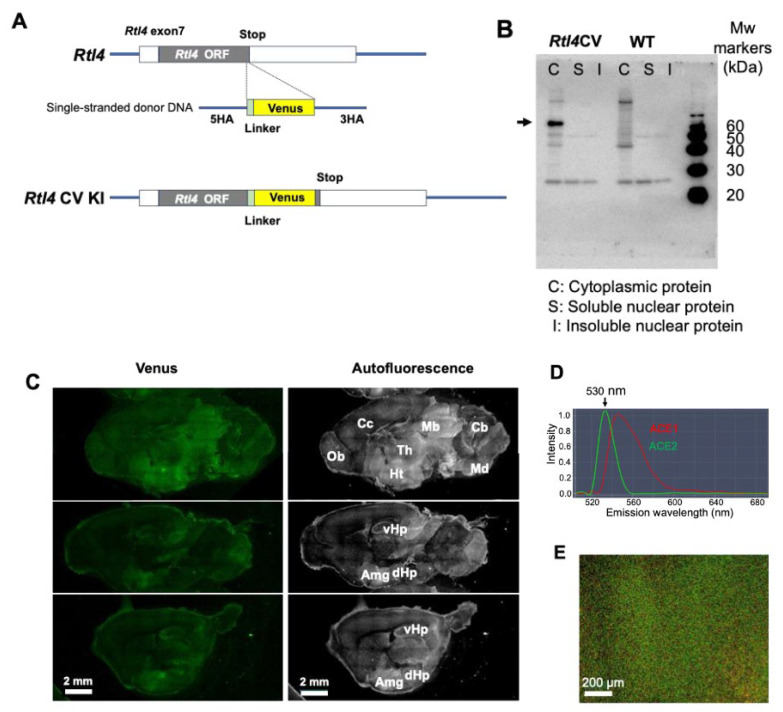

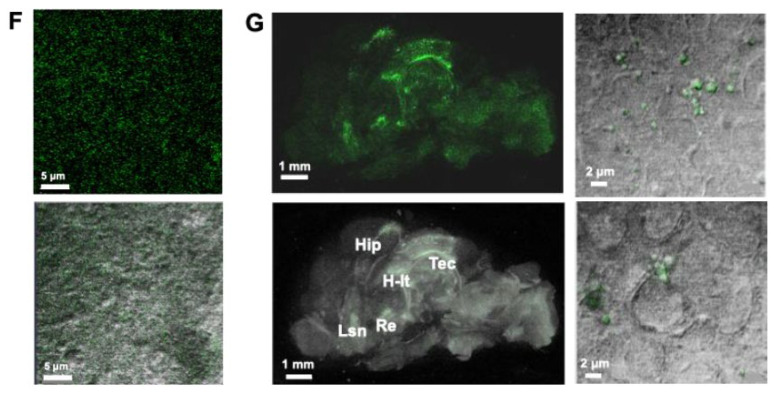
Detection of RTL4CV protein in the postnatal brain. (**A**) Production of the *Rtl4*CV knock-in mouse. Schematic representation of wild-type *Rtl4* and the modified genome structure of the *Rtl4* CV mouse. The mouse *Rtl4* open reading frame (gray box) is located on exon 7 (white box). The Venus coding sequence (yellow box) was inserted in front of the *Rtl4* stop codon together with a (GGS)x4 linker (light green box). See the details in Figure S1 and Table S1. (**B**) Immunoaffinity experiment of the RTL4-CV protein in the 2 w brain (indicated by an arrow). Immunoprecipitation was performed using an anti-GFP antibody. The estimated molecular weight of the RTL4CV protein is 61 kDa (RTL4 and Venus, 34 and 27 kDa, respectively). (**C**) Detection of RTL4CV in P15 brain. Left: Venus fluorescence image. Right: autofluorescence image. Amg: amygdala; Cb: cerebellum; Cc: cerebral cortex; dHP: dorsal hippocampus region; Ht: hypothalamus; Mb: midbrain; Md: medulla oblongata; Ob: olfactory bulb; Th: thalamus; vHp: ventral hippocampus region. (**D**,**E**) Venus signal detected by LSM880 confocal laser scanning microscopy. The Venus signal (530 nm) was detected as the second strongest peak (**D**) in the hypothalamus (**E**). ACE: Automatic Composition Extraction. (**F**) Dispersed dot-like signals observed in the hypothalamus. Top: Venus image. Bottom: Venus image merged with transmission image. See also Figure S6. (**G**) Granules of 1 μm containing RTL4CV in P2 brain. Several 1 μm granules were observed in the early postnatal periods. See also Figure S7. Top left: Venus image. Bottom left: merged image of autofluorescence and Venus. Right: granules in the hippocampus. Merged images of transmission and Venus. Hip: hippocampus; H-It: habenulo-interpedincular tract; Lsn: lateral septal nucleus; Re: thalamus nucleus reuniens; Tec: Tectal commissure.

*Rtl4*CV KI mice allowed for the detection of RTL4CV protein expression in the postnatal brain: it was detected mainly in the midbrain, hypothalamus, amygdala and hippocampus, as a widely distributed, presumably extracellular protein. To determine the location and dynamics of the RTL4 protein in the brain, we produced *Rtl4*CV KI mice (Figure 1A and Figure S1, and Table S1). Immunoaffinity experiments using an anti-Venus antibody revealed that the RTL4CV protein was detected in the 2 w brain at the expected molecular weight (61 kDa: RTL4 (34 kDa) + Venus (27 kDa)) (An arrow in Figure 1B).

**Figure 2:**
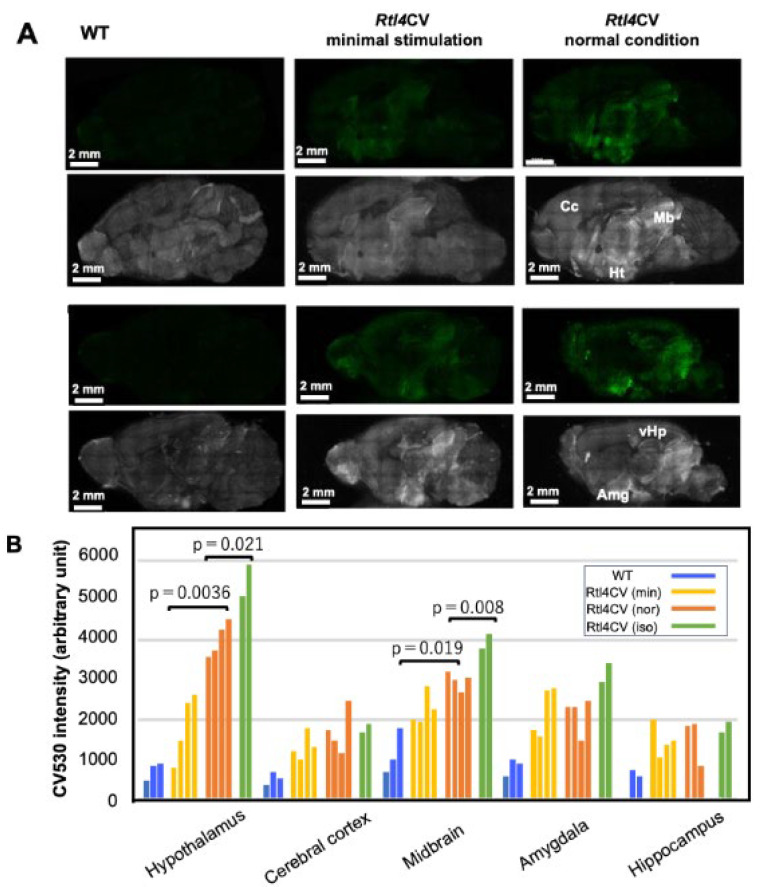

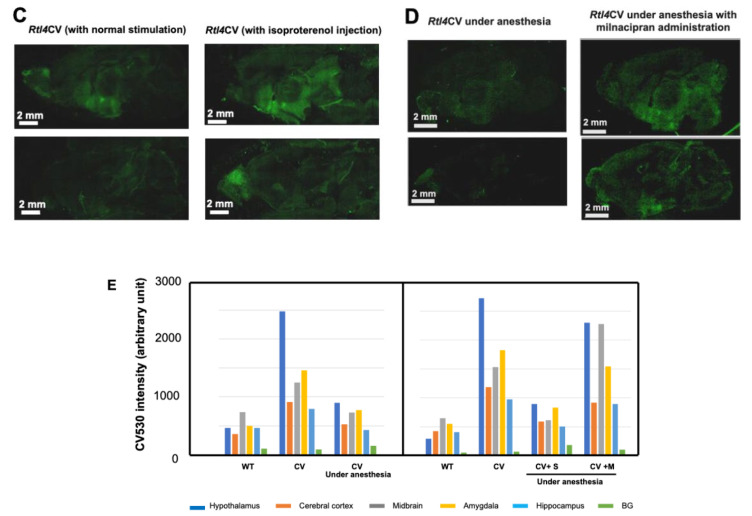
RTL4CV expression in the brain. (**A**) The effect of handling on RTL4CV protein expression in P21 brain. Venus and autofluorescence images of WT (left), *Rtl4*CV with “the minimal stimulation” (middle) and *Rtl4*CV in “the normal condition” (right). Top: inner side of the brain hemisphere. Bottom: brain slice of 1.5 mm width. (**B**) Measurement of Venus signal intensity at different parts of the P21 brain (see also Figure S10 for regional analysis method and Figure S11 for another example). Blue: WT; yellow: *Rtl4*CV with “the minimal stimulation” (min); orange: *Rtl4*CV under “the normal conditions” (nor). Green: *Rtl4*CV with isoproterenol cerebroventricular injection (iso, 10 μg/P21 mice). Each bar represents one P21 individual. The intensity of *Rtl4*CV in the hypothalamus and midbrain under “the normal conditions” (mean ± standard deviation (SD): 4004 ± 352 and 2970 ± 146) is significantly higher than that of *Rtl4*CV with “the minimal stimulation” (mean ± SD: 1805 ± 687 and 2231 ± 300) (*p* = 0.0036 and 0.019, two-tailed *t*-test), respectively. The intensity of *Rtl4*CV in the hypothalamus and midbrain is further increased after isoproterenol injection (mean ± SD: 5478 ± 381 and 3944 ± 175) (*p* = 0.021 and 0.008, respectively, two-tailed *t*-test). (**C**) Effect of isoproterenol on RTL4CV expression in P21 brain. Venus image of *Rtl4*CV under “the normal condition” (left) and *Rtl4*CV with isoproterenol injection (right). Top: inner side of the brain hemisphere. Bottom: brain slice of 1.5 mm in width. (**D**) Effect of anesthesia and milnacipran on RTL4CV intensity. Venus image of *Rtl4*CV under anesthesia (left) and anesthesia with milnacipran injection (right). Top: inner side of the brain hemisphere. Bottom: brain slice of 1.5 mm in width. (**E**) Graphic representation of (**D**)**.** Left: WT (P28), *Rtl4*CV (P25) and *Rtl4*CV (P25) under isoflurane anesthesia. Right: WT (P30), *Rtl4*CV (P27), *Rtl4*CV (P27) with saline injection (CV + S) and *Rtl4*CV (P27) with milnacipran injection (CV + M). After the *Rtl4*CV mice were anesthetized with isoflurane for 1 min, 10 μL of saline or milnacipran (total 167 μg) was administered intraventricularly, followed by additional anesthesia for 5 min. The signal intensity of the hypothalamus (blue), cerebral cortex (orange), midbrain, (gray) amygdala (yellow), hippocampus (light blue) and background (light green) are presented. See also Figure S12 for regional analysis and Figure S13 for another example.

The RTL4CV Venus signal was confirmed by confocal laser scanning microscopy (Figure 1C). The fluorescence from the RTL4CV protein was detected using a waveform with a peak at 530 nm emitted by Venus, calculated by the Automatic Composition Extraction (ACE) function (Figure 1D,E). The *Rtl4* mRNA expression levels were roughly estimated to be very low in the postnatal brain by qPCR (Figure S2). The *Rtl4/β-actin* ratio increased on around day 10 and peaked at around 3 w. This was more than 10-fold higher than the P1 level but still less than 1/4000 of the *β-actin* level (Figure S2). Although its mRNA level is very low in the P1 brain, Venus signals were detected in small but restricted regions, such as the midbrain and medulla oblongata (Figure S3). In the P5 brain, a strong Venus signal was detected in the midbrain, and a moderate signal was detected in the medulla oblongata and throughout the hypothalamus (Figure S4). In the P9 and P10 brain, the hypothalamic signal increased to the same level as the midbrain (Figure S5). After 2 w, the hypothalamus became the main site of expression, and amygdala and hippocampus signals were also detected in the P15 brain (Figure 1C,D). The hypothalamic signal increased until 3–4 w, and after that, it was maintained at a lower level than at 4 w, and the expression profiles remained the same. Most of the RTL4CV signal was detected as widely dispersed diffuse dots (Figure 1F and Figure S6), suggesting that it exists as a secretory protein, presumably in the extracellular space. An exception was the early postnatal period, such as P1 and P2; signals were detected in the midbrain and medulla oblongata in the P1 brain (Figure S3) and in the hippocampus, midbrain, lateral septal nucleus, thalamus nucleus reuniens and around the thalamus in the P2 brain (Figure 1G (left)), and the majority of these signals were in the form of presumed extracellular granules (1 µm in size) (Figure 1G (right) and Figure S7), suggesting that RTL4 may exist as a complex during this period. It is possible that some were intracellular, as there were a few cases with locations very close to the nuclei. In the WT brain, no intrinsic Venus signal was detected within ACE10 in any postnatal development stage, so the signal intensities were calculated using the control Venus waveform and treated as experimental background (BG) (Figures S8 and S9, see also Figure 2A,B).

### 2.2. RTL4CV Signal Intensity Varied in Response to Stressors, Environmental Changes and the Administration of Isoproterenol and Milnacipran

The patterns and intensities of RTL4CV varied in response to several factors related to NA. The previous *Rtl4* KO study suggested some relationship between RTL4 and NA. We hypothesize that RTL4CV is somehow responsive to NA, and this was supported by the finding that the intensity of RTL4CV signals was highly dependent on the experience of the *Rtl4*CV mice prior to observation. In the above analyses in Figure 1C and Figures S3–S5, the brain was observed after the mice were transferred from the breeding room to the laboratory (hereafter referred to as “the normal (or usual) condition (Nor)”). However, the RTL4CV signal intensity of the hypothalamus and midbrain was significantly lower when the mice were treated in the breeding room before transfer (hereafter referred to as “the minimal stress condition (Min)”) (Min: 1805 ± 687 and 2231 ± 300 vs. Nor: 4004 ± 352 and 2970 ± 146, *p* = 0.0036 and 0.019, two-tailed *t*-test), respectively (Figure 2A,B and Figures S10 and S11), suggesting that these regions are highly sensitive to various stimuli and/or stressors, such as differences in room brightness, ambient noise and vibration during cage transport. Even in the minimal stress condition, the signal intensity was variable, presumably due to inter-individual differences in sensitivity and/or slight differences in handling. Since NA plays an important role in arousal, attention, vigilance and stress responses, these results seem consistent with our hypothesis. An experiment with the intraventricular administration of isoproterenol (10 μg per P21 brain) (Iso) [26], an adrenergic receptor beta agonist that mimics the effects of NA, confirmed a significant increase (25%) in RTL4CV signal intensity in the hypothalamus and midbrain compared to those in “the normal condition” group (Iso: 5478 ± 381 and 3944 ± 175 vs. Nor: 4004 ± 352 and 2970 ± 146, *p* = 0.021 and 0.008, respectively, two-tailed *t*-test). (Figure 2B,C, see also Figures S10 and S11).

Furthermore, we also observed that isoflurane anesthesia of the *Rtl4*CV mice consistently decreased the signal intensity (Figure 2D (left), Figures S12 and S13 (left)), whereas it was maintained by the intraventricular administration of milnacipran (total 167 ng) [29] (Figure 2D (right), Figures S12 and S13 (right)). It is highly likely that the RTL4CV signal intensity depends on the arousal state and can be stabilized in the presence of NA by inhibiting NA reuptake, as NA is normally rapidly absorbed into neurons without milnacipran.

### 2.3. RTL4CV Is Expressed in Microglia and Responds to Isoproterenol

**Figure 3:**
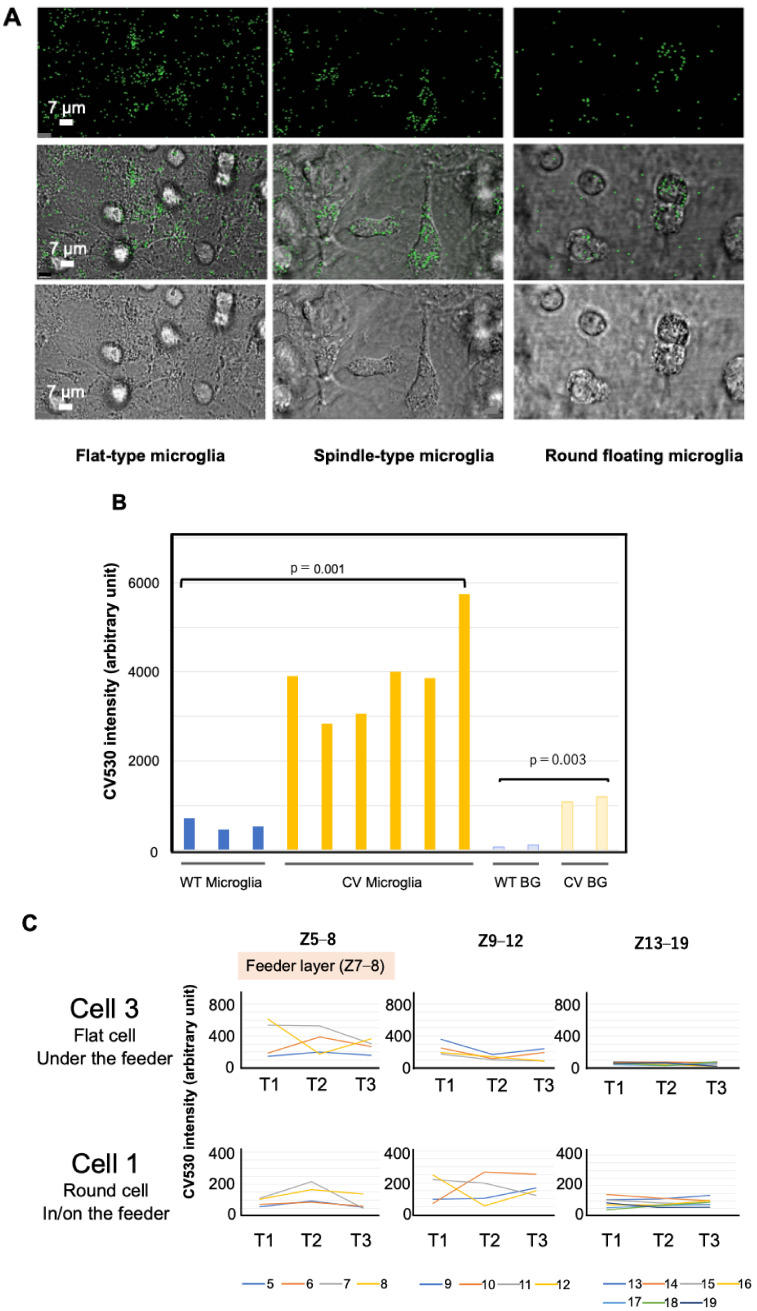
RTL4CV expression in cultured microglial cells. (**A**) Different types of microglia expressing RTL4CV in the mixed glial culture: (left) flat type, (middle) spindle type and (right) floating round type (see also Figure S14). Top: Venus images. Middle: merged images of transmission and Venus. Bottom: transmission images. (**B**) Measurement of Venus signal intensity of microglial cells isolated from WT (blue) and *Rtl4*CV (yellow) mice (left) and that of culture media from WT (light blue) and Rtl4CV (pale yellow) mice (right). The intensity of *Rtl4*CV microglia (3901 ± 648) is significantly higher than that of WT microglia (567 ± 96) (*p* = 0.001, two-tailed *t*-test). The same is true for the culture media (CV: 1162 ± 106, WT: 53.5 ± 21.5) (*p* = 0.003, two-tailed *t*-test). (**C**) Rapid secretion of RTL4CV in response to isoproterenol administration. Each of the 3D-section intensity data (at 0.5 μm intervals, a total of 19 Z positions) from the mixed glial culture dishes was obtained in the time-lapse experiment (90 s interval). Isoproterenol (20 μM) was added to the culture media during the 90 s interval between T1 and T2. Therefore, the T2 data were obtained 30 s after administration. As shown in the top label, the astrocyte feeder layers were located at Z7–Z8, and the microglial cells were located from Z8 (within the feeder) to Z11–Z12 (on the feeder) according to their morphology. The signals at the Z position numbers Z5–Z8, Z9–Z12 and Z13–19 are displayed. The results of the two microglial cells (No. 1 and 3 in Figure S15) are shown. The results of Z1–Z4 (bottom side) and BG are shown in Figure S15.

Cultured cell experiments confirmed that RTL4CV is expressed in microglia and that RTL4CV secretion is responsive to isoproterenol. Since the distribution of the RTL4CV protein as a dispersed secretory protein is similar to that of the RTL5 and RTL6 proteins secreted by microglia [38], we tested whether microglia express RTL4CV by performing mixed glial culture experiments on glia isolated from neonatal KI mouse brains (P0 or P1) [38,39,46]. Under this in vitro condition, several types of microglia, such as flat, spindle-shaped and floating round cells, were observed within, on and above astrocyte feeder cells, respectively, by immunofluorescence staining with an anti-Iba1 antibody (Figure S14 and Supplementary Movie S1). The RTL4CV signal was detected in all types of microglia (CV: 3901 ± 648 vs. WT: 567 ± 96, *p* = 0.001, two-tailed *t*-test) but not the astrocyte feeder cells (Figure 3A,B). Interestingly, the RTL4CV signal intensity was also higher in the culture medium of *RTL4*CV than that of the control WT microglia (CV: 1162 ± 106, WT: 53.5 ± 21.5, *p* = 0.003, two-tailed *t*-test), suggesting that RTL4CV is secreted into the culture medium under this condition (Figure 3B). Although the possibility of RTL4CV expression in neurons and/or oligodendrocytes cannot be completely excluded, these results demonstrated that the RTL4C protein is expressed in microglia and secreted into the brain.

Furthermore, rapid changes in signal intensity were observed in and around the microglia immediately after isoproterenol (20 μM) administration into the culture media [27]. We analyzed the results of the three-dimensional (3D) time-lapse experiments in the mixed glial culture before (T1) and after (T2 and T3) isoproterenol administration (Figure 3C). The astrocyte feeder layers were located at Z7–Z8, and the microglial cells within the feeder were located at approximately Z8, while those on the feeder were located at approximately Z11–Z12. The signal intensities of the microglial cells decreased rapidly (within 30 s) after isoproterenol administration (e.g., at Z8 in Cell 3 and at Z12 in Cell 1), whereas those of the area beneath the microglial cells increased in the opposite manner (e.g., at Z6 in Cell 3 and at Z10 in Cell 1). The intensity of the other Z positions (e.g., Z1–4 and Z13–19 in Cell 3 and Cell 1) remained unchanged (Figure 3C and Figures S15). These results suggest that the microglial cells secreted RTL4CV into the media in response to isoproterenol.

## 3. Discussion

In this work, using KI mice carrying a gene that fuses endogenous *Rtl4* with *Venus* to produce the RTL4CV protein, we demonstrated that *Rtl4* is a fourth microglial gene among the RTL/SIRH genes and that the RTL4 protein is secreted in the developing brain and its secretion is enhanced by various stimuli, possibly related to NA.

### 3.1. RTL4, a Causative Gene in ASD, Is a Microglial Gene

*RTL4* has been implicated in ASD [14,15,16], and the *Rtl4* KO mice exhibited behavioral abnormalities, such as increased impulsivity, reduced adaptation to novel environments and impaired short-term spatial memory, as well as a low recovery rate of NA in the frontal cortex [17]. In this study, we demonstrated that a causative ASD gene, *Rtl4,* is a microglial gene that responds to NA signaling in the brain. The in vitro experiments demonstrated that RTL4CV is expressed in microglia, not astrocytes, and secreted into the culture media and suggested that RTL4CV secretion responds rapidly to isoproterenol administration. The dynamics of the RTL4CV protein in the postnatal brain demonstrated in this study, such as its possible dependence on arousal state and highly sensitive response to stress/environmental changes, including the response to isoproterenol and milnacipran, could provide critical information in elucidating the etiology of ASD. As the NA response to stressors is likely to be reduced under anesthesia, RTL4 may also be a good indicator of arousal state and/or NA secretion in certain brain regions.

It has been shown that stress and the associated regulation of corticotropin-releasing factor (CRF) and opioid hormones affect the development of the NAergic system in the brain, and that this adaptive stress response promotes behavioral flexibility [47,48,49]. Since RTL4 secretion is induced by various stressors, such as the handling and transfer of mice from the breeding room to the laboratory (Figure 2A–D), the lack of this response from postnatal to adult life may explain why the *Rtl4* KO mice exhibited reduced adaptability to a new environment [15], possibly due to impaired development of the NAergic system. The RTL4CV signal was maintained after the administration of milnacipran (Figure 2D), suggesting that RTL4CV intensity is dependent on the presence of NA, although the mechanism by which the RTL4CV signal is stabilized by NA remains unclear. Is it possible that secreted RTL4 forms a complex with NA (see last section) or that secreted RTL4 is somehow involved in the reuptake of NA in neurons? Assuming that RTL4 has the ability to support the NA reuptake process, this may be consistent with the previous findings that *Rtl4* KO exhibited increased impulsivity and slow recovery of NA levels in the prefrontal cortex [17] because NAT is responsible for the reuptake of most of the secreted NA back to the presynaptic noradrenergic neurons [28]. The resulting low level of NA in the NA neurons may be related to the impaired short-term spatial memory of the *Rtl4* KO mice [17,50]. Taken together, RTL4 is a novel and important microglial factor in the brain’s response to NA and may serve as an important therapeutic target in ASD, although further studies are needed to elucidate the exact function of the secreted RTL4 protein in the NA reuptake process.

### 3.2. Importance of RTL4 in the Developing Brain and Its Relationship with the LC-NA System

What is the role of RTL4 secretion in the brain? Since NA reportedly shapes the properties of NAergic neuronal networks in the developing brain [30,31], it is likely that an impaired NA response during the postnatal period interferes with the normal development of NAergic neuronal networks. Importantly, the timing and location of RTL4 expression as well as its induction closely correspond with critical periods and regions in brain development. Learning to recognize the maternal odor is critical for the survival of infants in order to receive proper maternal care, known as infant attachment learning. Incidentally, β-ARs play an important role in infant attachment learning, and the absence of α2-AR activity during this period is also considered important [31]. During this early postnatal period, RTL4CV was present mostly as 1 µm, presumably extracellular granules in the hippocampus, midbrain, lateral septal nucleus, thalamus nucleus reuniens and around the thalamus (Figure 1G). The hippocampus, which is the well-known center of memory and the lateral septal nucleus, with its abundant inputs from neocortical and allocortical regions, is ideally positioned to integrate perceptual and experiential signals and plays a critical role in emotionality, social behavior and feeding processes through neural connections with the hippocampus, midbrain, thalamus nucleus reuniens and hypothalamus [51,52]. It is therefore highly likely that RTL4 plays an important role in this process.

The intrinsic RTL4CV signal was very strong around P10 (Figure S5). After P10 in rats, neonates acquire aversion and fear learning [31]. The inhibitory effect of α2-AR is activated subsequent to P10 in rats and is known to play an important role in the subsequent formation of the NAergic neuronal network. The interaction between LC activity and NA signaling in the hypothalamus and hippocampus, as well as the corresponding activity in the amygdala, has been suggested to be important for fear learning in early development. Since amygdala expression was observed in the P10 mouse brain under “the normal conditions” (Figure S5), it is possible that RTL4 also takes part in this process.

The RTL4CV signal peaks at 2–4 weeks (2–4 w) (Figure 1C and Figure 2A). In rats, robust synaptogenesis in the noradrenergic pathway occurs between P10–15 and P20–30. α-ARs are present in newborns, but their number increases at most sites after birth, peaking at around P15 for α2-ARs [53] and between P15 and 20 for α1-ARs [31,54]. In the rat cerebral cortex, β-AR density increases gradually from P5, reaching values higher than adult levels by 2–3 w and then maintaining adult levels for several months [31]. This is in good agreement with the RTL4 profiles in these mice (Figure 1C, Figure 2A and Figure S2). It is likely that various different stimuli are able to induce RTL4 expression in different brain regions so as to somehow promote the new NAergic neuronal network (Figure 2A–D).

### 3.3. Rtl4 Is the Fourth RTL/SIRH Gene Identified in Eutherian Microglia

RTL/SIRH genes are genes derived from *Metaviridae*, retrovirus-like retrotransposons, by three independent domestication events, resulting in therian-specific *PEG10*, eutherian-specific *RTL1* (also known as *PEG11*) and *RTL3-9* (*SIRH3-11*), respectively [1,2,3,4,5,6,7,8,9,10,11]. They all encode Gag-like proteins, but *PEG10*, *RTL1* and *RTL3* (also known as *SIRH9*) also encode a portion of Pol-like protein. They are good examples of exaptation proposed by Gould and his colleagues [55,56], because each of the ten RTL/SIRH genes has a unique, essential and/or important function in the placenta and/or brain [8,10]. *PEG10*, *RTL1* and *LDOC1* (also known as *RTL7* or *SIRH7*) play essential/important roles in both the placenta [5,57,58,59,60,61] and in the brain as neuronal genes, along with *RTL8A*, *B* and *C* (also known as *SIRH5*, *6*, *4*) [62,63,64,65,66,67], whereas *RTL5* (also known as *SIRH8*), *RTL6* (also known as *SIRH3*) and *RTL9* (also known as *SIRH10*) play roles in the innate immune system as microglial genes in the brain against viruses, bacteria and fungi by removing dsRNA/ssDNA, LPS and zymosan, respectively [38,39].

Thus, *RTL4* is the fourth microglial gene among the RTL/SIRH genes [10], suggesting that these four RTL genes contribute to eutherian-specific microglia. What is the role of RTL4 in microglia? Apparently, RTL4 responds to psychological stressors via NA, whereas RTL5, RTL6 and RTL9 respond to physiological stressors, i.e., various pathogens, suggesting that each of these RTL genes plays a distinct role in the brain’s stress responses in a eutherian-specific manner. The retroviral GAG from which each RTL/SIRH gene is derived encodes molecules that can self-assemble. RTL5 and RTL6 form large extracellular particles that bind specific PAMPs, as if taking advantage of this property of GAG [38]. One possibility is that RTL4 supports the NA reuptake response, as discussed above, by forming a sort of RTL4-NA complex similar to the RTL5-dsDNA and RTL6-LPS complexes.

In the early postnatal period, similarly to RTL5 and RTL6 [38], RTL4 was present as 1 µm, presumably extracellular granules in specific regions where new neuronal networks are thought to form (Figure 1G (right) and Figures S7) [51,52]. Although it remains unclear what components are contained within these granules, it is interesting to speculate that RTL4 recognizes some damage-associated molecular patterns (DAMPs) and acts as a scavenger of these substances to maintain a healthy brain environment during this period because it is possible that cellular debris is generated during NA-induced network remodeling [40,68,69]. This situation is similar to recognition receptors (PRRs), such as Toll-like receptors (TLRs) and cytoplasmic NOD-like receptors (NLRs), which recognize both PAMPs and DAMPs to orchestrate an inflammatory response [70,71]. Regardless of its relationship with NA per se, it seems likely that eutherians can improve their use of NA with the acquisition of *RTL4* and that *RTL4* defects can lead to the development of ASD.

As microglia have recently attracted considerable attention as an important factor in the pathogenesis of not only ASD but also other neurodevelopmental and neuropsychiatric disorders [40,41,42,43,69,72,73,74], further experiments are needed to elucidate the biochemical function of RTL4, as well as other, as yet unidentified microglia-specific genes, in the etiology of these neurodevelopmental and neuropsychiatric disorders, including ASD. This study provides evidence not only for the role of a novel microglial gene involved in ASD but also for the unexpected contribution of a retrovirus-derived acquired gene to the evolution of the eutherian brain, along with other neuronal genes, such as *PEG10*, *PEG11*/*RTL1* and *RTL8A-C* [62,63,64,65,66,67], and microglial genes, such as *RTL5*, *RTL6* and *RTL9* [38,39].

Although the human and mouse brains have the same basic structure, it is known that there are differences not only in the development of the cerebral cortex but also in the presence of human-specific brain components and in the expression of genes involved in neural regulation and development, such as serotonin receptors and glutamate receptor subunits [75,76]. Therefore, it is not possible at this time to predict the extent to which the mouse models will reflect human ASD. However, β-AR antagonists are currently being used in clinical trials to improve core symptoms and anxiety in patients with ASD [77], suggesting that NA plays an important role in the pathogenesis of ASD. As *RTL4* is a newly acquired gene in eutherians, it is possible that it shares common properties with NA signaling in mice and humans, at least at the level of protein function, and we hope that the findings described here will help elucidate the pathogenesis of ASD.

## 4. Materials and Methods

### 4.1. Mice

All of the animal experiments were reviewed and approved by the Institutional Animal Care and Use Committees of Tokai University (245001, 1 April 2024) and Tokyo Medical and Dental University (TMDU) (A2022-57A, 23 March 2022) and were performed in accordance with the Guidelines for the Care and Use of Laboratory Animals of Tokai University and TMDU. Animals were allowed access to a standard chow diet and water ad libitum, and were housed in a pathogen-free barrier facility with a 12 h light:12 h dark cycle. C57BL/6N (B6) mice were used to generate *Rtl4*CV mice and as controls throughout the study. 

### 4.2. Generation of the Rtl4CV Mice

*Rtl4*CV mice were generated as previously described with minor modifications [38]. Genomic DNA near the stop codon of the *Rtl4* gene was cleaved using Cas9 and a guide RNA targeting 5′-AACGTTCTAGAACTCCAGCA-3′. A plasmid vector was constructed to introduce DNA encoding most of the C-terminal amino acids of *Rtl4* located downstream of the Cas9 cleavage site, a 12-amino acid linker ((GGAGGATCA)x4) and the Venus protein in the Cas9 cleavage site upstream of the *Rtl4* stop codon so that RTL4 and Venus were expressed as a fusion protein connected to the linker. The vector also had homology arms of 1500 bases at the 5′ and 3′ ends of the introduced sequence, and the codon encoding arginine 301 was mutated from AGG to AGA to prevent re-cleavage by Cas9 after genetic recombination. The single-stranded DNA was synthesized as a mutation donor, as previously described [34], and used to generate the genetically modified mice. Briefly, double-stranded DNA was amplified using the primer pair (5′ to 3′) TGCGTCCACTACCAAAGGAT and pre-phosphorylated GAGGAGGGGCTATCTTTCAAAC, and its 5′-phosphorylated strand was digested with lambda exonuclease.

Founder mice were genotyped using the three primer pairs (5′ to 3′) CCAGATTTGATCACTCAGTGC/TGTTGTGGCGGATCTTGAAG, AGCAGCACGACTTCTTCAAG/CTCTTGAAGCTGATTGGTCC and CCAGATTTGATCACTCAGTGC/CTCTTGAAGCTGATTGGTCC. Two founder mice had unexpected mutations in the 3′UTR of *Rtl4*, a 12-base pair insertion and a 1-base pair deletion in founder #10, and a 2-base pair deletion in #13. These founders were used to generate 2 *Rtl4*CV lines, and their progeny were used for further experiments.

### 4.3. Immunoprecipitation and Western Blotting

The postnatal brain (2 w) was powderized in liquid N_2_ using a multi-bead shocker (MB1050, YASUI KIKAI, Osaka, Japan), and the “Cytoplasmic Fraction”, “Soluble Nuclear Fraction” and “Insoluble Nuclear Fraction” were obtained using the LysoPure^TM^ Nuclear and Cytoplasmic Extraction Kit (FUJIFILM, Code No. 295-73901, Tokyo, Japan). The powder samples of the WT and *Rtl4*CV brains (86.7 mg and 67.8 mg, respectively) were dissolved in 1000 µL of Nuclear Fractionation Buffer on ice for 10 min. After centrifugation (500× *g*, at 4 °C) twice for 10 min, the supernatants were mixed and used as the “Cytoplasmic Fraction”. The pellets were resuspended in 500 µL of Nuclear Fractionation Buffer and mixed vigorously. After centrifugation (500× *g*, at 4 °C) for 10 min, the supernatant was discarded and the pellet was resuspended in 500 µL of Nuclear Extraction Buffer on ice for 30 min with vigorous mixing at 10 min intervals. After centrifugation (20,000× *g*, at 4 °C) for 10 min, the resulting supernatant was used as the “Soluble Nuclear Fraction”. The pellet was resuspended in 500 µL of Nuclear Extraction Buffer with vigorous mixing. After centrifugation (20,000× *g*, at 4 °C) for 10 min, the supernatant was discarded and the pellet was resuspended in 500 µL of Nuclear Extraction Buffer with vigorous mixing. After centrifugation (20,000× *g*, at 4 °C) for 10 min, the supernatant was discarded and the pellet was resuspended in 400 µL of SDS Lysis Buffer with vigorous mixing and sonicated until the pellet was no longer visible. After centrifugation (20,000× *g*, at 4 °C) for 10 min, the supernatant was used as the “Insoluble Nuclear Fraction”.

Each supernatant was mixed with 20 µL of anti-GFP (RatIgG2a), Monoclonal (GF090R), CC, Agarose Conjugate (NACALAI TESQUE, Tokyo, Japan) and incubated overnight at 4 °C. The agarose beads were washed four times with 500 µL of RinseBuffer, 50 mM Tris-HCl (pH 8.0) and 150 mM NaCl at 4 °C. Then, the beads were incubated with 30 µL of SDS sample buffer and incubated at 95 °C for 5 min. Gel electrophoresis was applied to the samples using a 10% acrylamide gel. Western blot analysis was performed using a standard protocol. After blotting on a Hybond-P (GE Healthcare, Chicago, IL, USA) membrane, the RTL4CV protein was detected with an ECL Prime Western Blotting Detection kit (GE Healthcare) using an anti-GFP antibody (MBL, Code No. 598) and an anti-Rabbit Goat Immuno-globulins/HRP (DAKO, P0160) as the 1st and 2nd antibodies. Signals were detected with an AE-9300 Ez CaptureMG (ATTO, Tokyo, Japan).

### 4.4. Imaging Using Confocal Laser Scanning Fluorescence Microscopy

Fresh brain and brain slices (1.5 to 2 mm in depth) from *Rtl4*CV KI mice were used for analysis with a ZEISS LSM880 (ZEISS, Oberkochen, Germany) without fixation. More than 100 brain samples from P1 to 8 w mice were analyzed. The samples were observed using a Plan-Apochromat lens (10×, numerical aperture = 0.45, M27, ZEISS) and a C-Apochromat lens (63× numerical aperture = 1.2 Water, ZEISS). Tiling with lambda-mode images was obtained using the following settings: pixel dwell: 1.54 μs; average: line 4; master gain: ChS: 1250, ChD: 542; pinhole size: 33 µm; filter 500–696 nm; beam splitter: MBS 458/514; and lasers: 514 nm (Argon 514), 0.90%. For the tiling-scan observations, the tiling images were captured as follows: tiles: 84; overlap in percentage: 10.0; tiling mode: rectangular grid; and size: 15,442.39 µm in x and 9065.95 µm in y. Spectral unmixing and processing of the resulting images were performed using ZEN imaging software (version 2.3 SPI, Carl Zeiss Microscopy, Jena, Germany). The spectrum of the Venus proteins (maximum peak-emission fluorescence wavelength: 528 nm) was detected only in the *Rtl4*CV samples and not in the wild-type control samples. ZEN Blue edition ver. 3.9 (ZEISS) was used to measure and correct the average intensity of fluorescent molecules and the average tissue and extracellular steady-state background fluorescence intensity from 2D, 3D and 4D tissue and cell fluorescence images. Briefly, the raw images were averaged to remove noise, and each component image (RTl4CV530, AF530, etc.) was acquired by spectral analysis. The target region areas (the cortex, hypothalamus, amygdala, midbrain, hippocampus, etc.) of the acquired images were manually identified for validity, and only the target areas were analyzed. Evaluation images were acquired using IMARIS ver. 6.1 (BitPlane, Concord, MA, USA).

The 3D time-lapse images were obtained under the following conditions. Microglial cells were cultured in 35 mm glass-bottom dishes in a humidified incubator with 5% CO_2_ at 37 °C using the Signal-top incubation system S1 (ZEISS, Germany). Time series: 90 s interval, 11 frames. After obtaining the first frame of the mixed glial culture, isoproterenol (20 μM) was added to the culture media over a 90 s interval, and the second to eleventh frames were obtained. The first 3 frames (first frame: untreated; second and third frames: after drug administration) are shown (Figure 3C and Figures S9). Z-stack: 0.5 μm steps 28 slices (13.713 μm). Scaling (per pixel): 0.26 μm × 0.26 μm × 0.51 μm. Image size (pixels): 512 × 512. Bit depth: 16 bit. Objective lens: C-Apochromat 63×/1.2 W Korr M27. Filters: 499–695. Beam splitter: lambda. MBS: MBS458/514. MBS-InVis: plate. DBS1: mirror. Laser: Ar 514 nm, 0.9%. Scan mode: line sequential. Pixel time: 1.54 μs. Line time: 30.00 μs. Frame time: 1.00 s.

### 4.5. Primary Mixed Glial Culture

Primary mixed glial cultures were prepared according to the protocol of Lian et al. with modifications [46]. Briefly, P0–P2 brains were homogenized in phosphate-buffered saline (PBS) supplemented with trypsin and DNase (final concentration: 0.05% and 0.1 mg/mL, respectively) by repeated pipetting 10 times and incubated in a 37 °C water bath for 5 min, and this process was repeated 3 times. After the addition of Dulbecco’s modified Eagle medium (DMEM) (Thermo Fisher Science, Gibco^TM^ Catalog No. 11995065, Waltham, MA, USA) along with 10% fetal bovine serum (FBS) (Thermo Fisher Science, Catalog No. 10437028), the debris was removed through a cell strainer (EASYstrainer, mesh size: 100 μm, Greiner, Cat. No. 542000, Kremsmunster, Austria). After centrifugation (400× *g*, at 4 °C) for 5 min, the supernatants were aspirated and the pellet was resuspended with 5 mL of warmed culture medium. After determining the cell density, the mixed glial cells were plated into 35 mm glass-base dishes (IWAKI, Code No. 3910-035, Osaka, Japan) or 25 cm^2^ flasks (Canted Neck, Corning, Cat. No. 430639, New York, NY, USA) and incubated in a CO2 incubator with 5% CO_2_ and 100% humidity at 37 °C. Media were changed the next day and every 3–4 days thereafter. More than 10 and 40 microglial samples from WT and *Rtl4*CV mice were analyzed, respectively.

### 4.6. Isoproterenol and Milnacipran Administration

Isoproterenol hydrochloride (FUJI FILM, Code No. 553-69841) was dissolved in 0.9% sodium chloride saline at a concentration of 1 mg/mL just before the experiment. Based on a previous report by Yabuuchi et al. [26], a 10 μL aliquot (10 μg/P21 mice) was administered intracerebroventricularly with an 18G needle syringe, or 20 μM (final concentration) of isoproterenol was added to the culture medium according to a previous report by Tomozawa et al. [27]. Milnacipran hydrochloride (Tokyo Chemical Industry (TCI), Code No. M2133, Tokyo, Japan) was dissolved in 0.9% sodium chloride saline at a concentration of 50 mg/mL and stored at −80 °C. A 10 μL aliquot (167 ng) was administered intracerebroventricularly with an 18G needle syringe according to a previous report by Bagchi et al. [29]. Mice were anesthetized using an isoflurane anesthesia machine, SN-487-OT (SINANO MFG Co., Ltd., Tokyo, Japan), under conditions of 4% isoflurane for 1 min, and then saline or milnacipran was injected intracerebroventricularly under 2.5% isoflurane, and the mice were further anesthetized for 5 min. Three experiments were performed with the administration of isoproterenol and milnacipran, respectively, and control isoflurane anesthesia.

## 5. Conclusions

We have demonstrated that a causative ASD gene, *RTL4*, is a microglial gene, and that RTL4 is present as a secretory protein, presumably in the extracellular space of the postnatal brain. RTL4 expression is well correlated with the developing NAergic neuronal network in the postnatal period, involving aspects such as infant attachment learning, aversive and fear learning and robust synaptogenesis, which are associated with an increase in α1-, α2- and β-adrenergic receptors. Its secretion is responsive to NA signaling and a variety of stimuli, suggesting that it may serve as an important therapeutic target in ASD via its possible involvement in the NA reuptake process. *RTL4* is the fourth microglial gene among the eutherian-specific RTL/SIRH genes, which are derived from a certain retrovirus belonging to *Metaviridae*. This study provides evidence not only for the role of a novel microglial gene in ASD but also for the unexpected contribution of a retrovirus-derived acquired gene to the evolution of the eutherian brain, along with other neuronal genes, such as *PEG10*, *PEG11/RTL1* and *RTL8A-C*, and microglial genes, such as *RTL5*, *RTL6* and *RTL9*.

## Data Availability

Data can be made available upon request by the corresponding author. *Rtl4*CV mice are available from the RIKEN BioResource Center (Tsukuba, Japan), along with *Rtl4* KO, *Rtl5*CmCherry (CmC), *Rtl5* KO, *Rtl6*CV, *Rtl6* KO, *Rtl9*CmC and *Rtl9* KO mice.

## Supplementary Materials

The supporting information can be downloaded at https://www.mdpi.com/article/10.3390/ijms252413738/s1.
