# Supplementary material for: RTL4, a Retrovirus-Derived Gene Implicated in Autism Spectrum Disorder, Is a Microglial Gene That Responds to Noradrenaline in the Postnatal Brain
Source: Int J Mol Sci. 2024 Dec 23;25(24):13738. doi: 10.3390/ijms252413738 (PMC11678650; doi:10.3390/ijms252413738)
Supplement: Supplementary file 1 [file ijms-25-13738-s001.zip › ijms-3301339-supplementary.pdf]

A

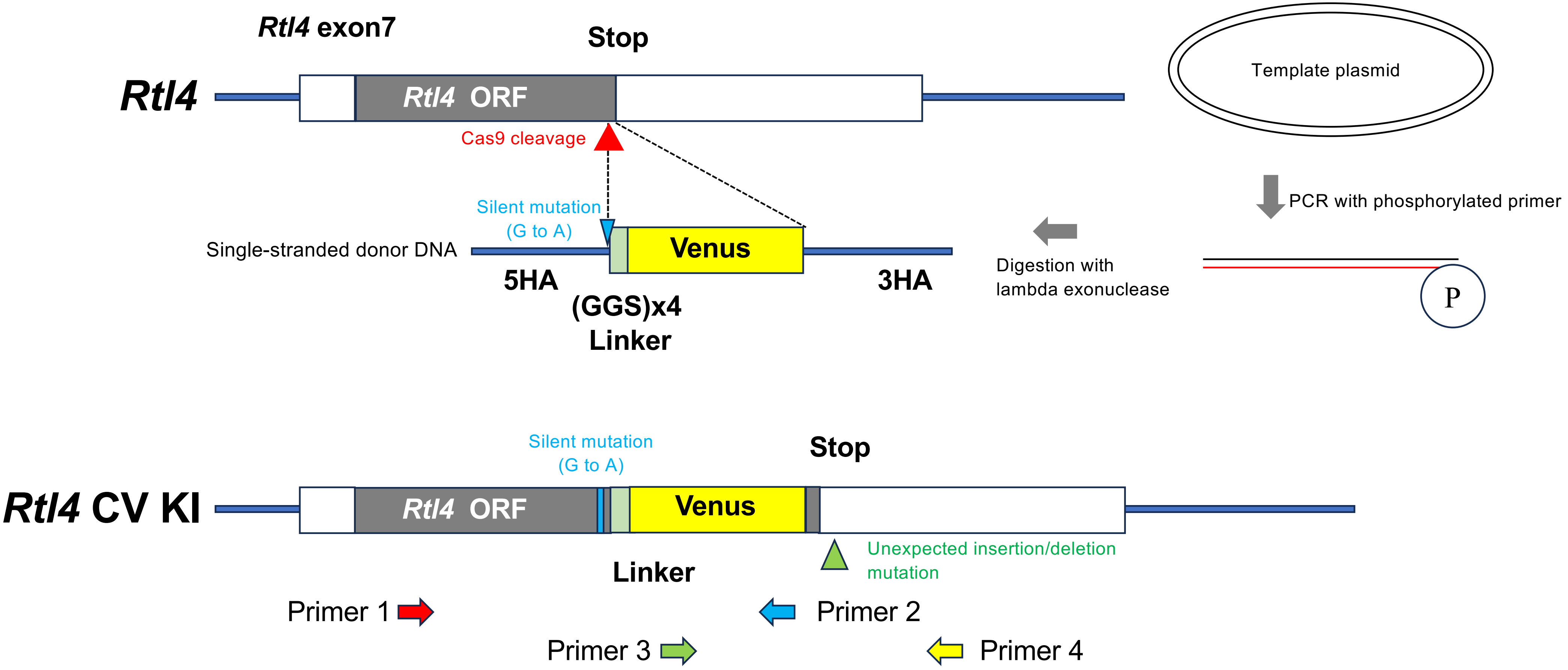

Fig. S1-1 Generation of the *Rtl4*CV mice

B

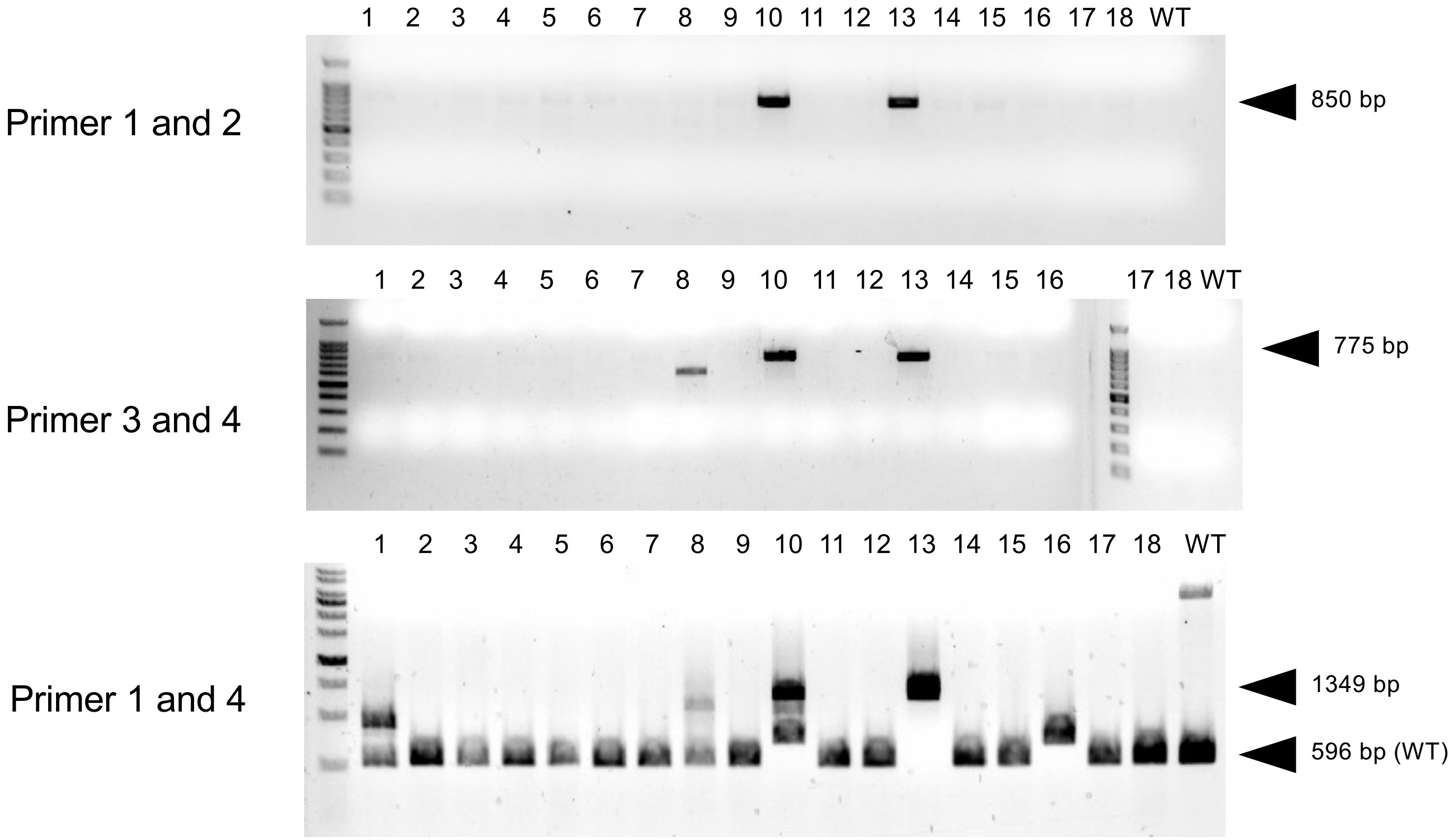

Fig. S1-2 Generation of the *Rtl4*CV mice

C

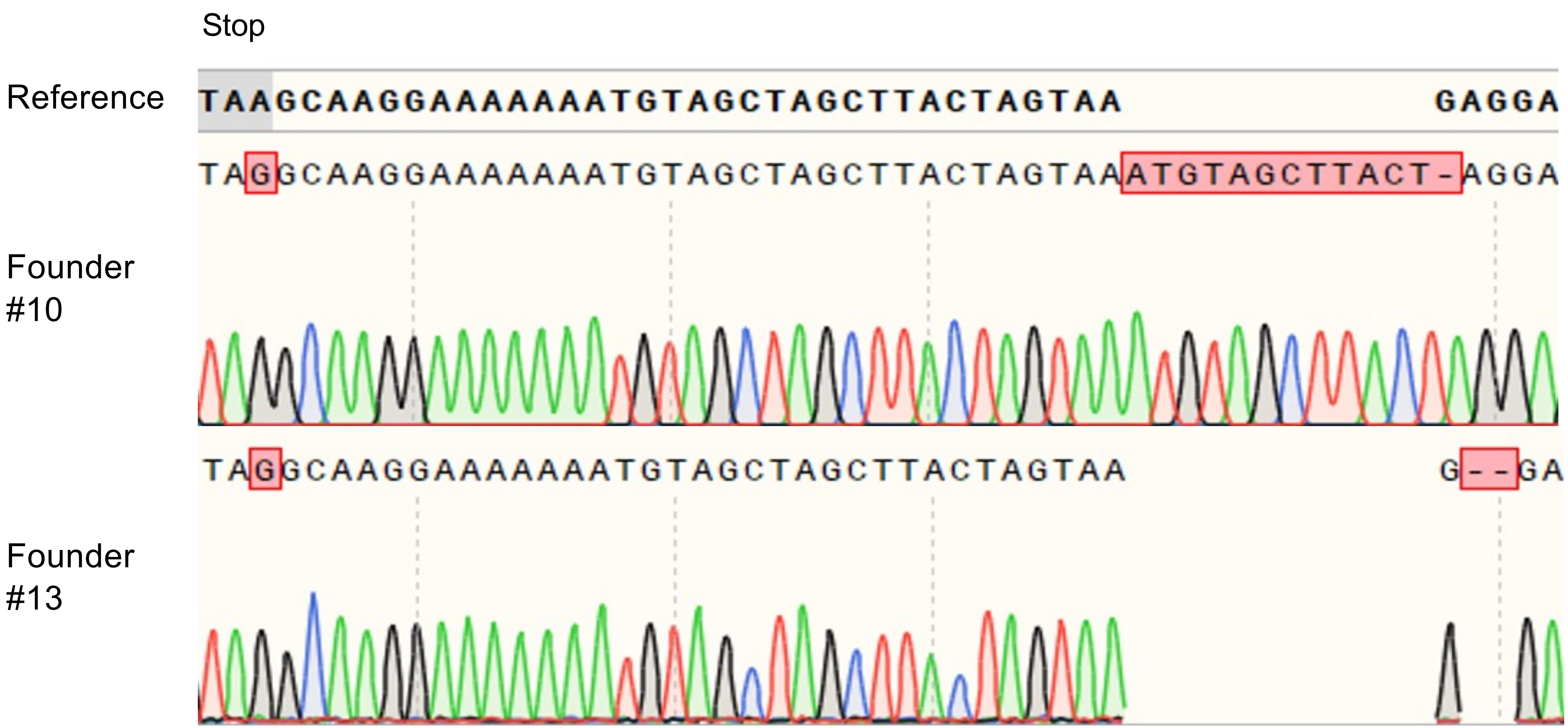

Fig. S1-3 Generation of the *Rtl4*CV mice

Fig. S1 Generation of the *Rtl4*CV mice

A. Schematic representation of the genome modification in the *Rtl4*CV mice

The Venus coding sequence was introduced into the Cas9 cleavage site upstream of the *Rtl4* stop codon together with a (GGS)x4 linker to express the RTL4-Venus fusion protein. To prevent recleavage of the *Rtl4*CV mouse genome after the genetic recombination, an A to G mutation was introduced in the codon of arginine 301 (AGG to AGA). The single-stranded donor DNA for gene modification was prepared by phosphorylated primer-mediated PCR, and the phosphorylated strand digestion with lambda exonuclease (Table S1). The donor DNA has 5' and 3' homology arms of 161 and 221 bases, respectively. The Cas9 cleavage site was targeted near the *Rtl4* stop codon. The white squares show the 5' and 3' UTR in the *Rtl4* coding exon, and the gray square shows the *Rtl4* coding sequence.

B. Genomic PCR analysis of the *Rtl4*CV founder mice

The primer positions are shown in A (see also Table S1). The founder number and wild type (WT) as a control are shown above each panel. The expected product sizes of the *Rtl4*CV allele and WT are shown to the right.

C. Genome sequences of the *Rtl4*CV founder mice

Detailed DNA sequences are shown for founder #10 and #13 with the reference genome sequence. Founder #10 and #13 have additional insertion/deletion mutations in the 3’UTR of *Rtl4*. “Stop” indicates the position of the stop codon located downstream of the Venus sequence in the *Rtl4*CV. The sequencing data were analyzed using SnapGene software.

’.

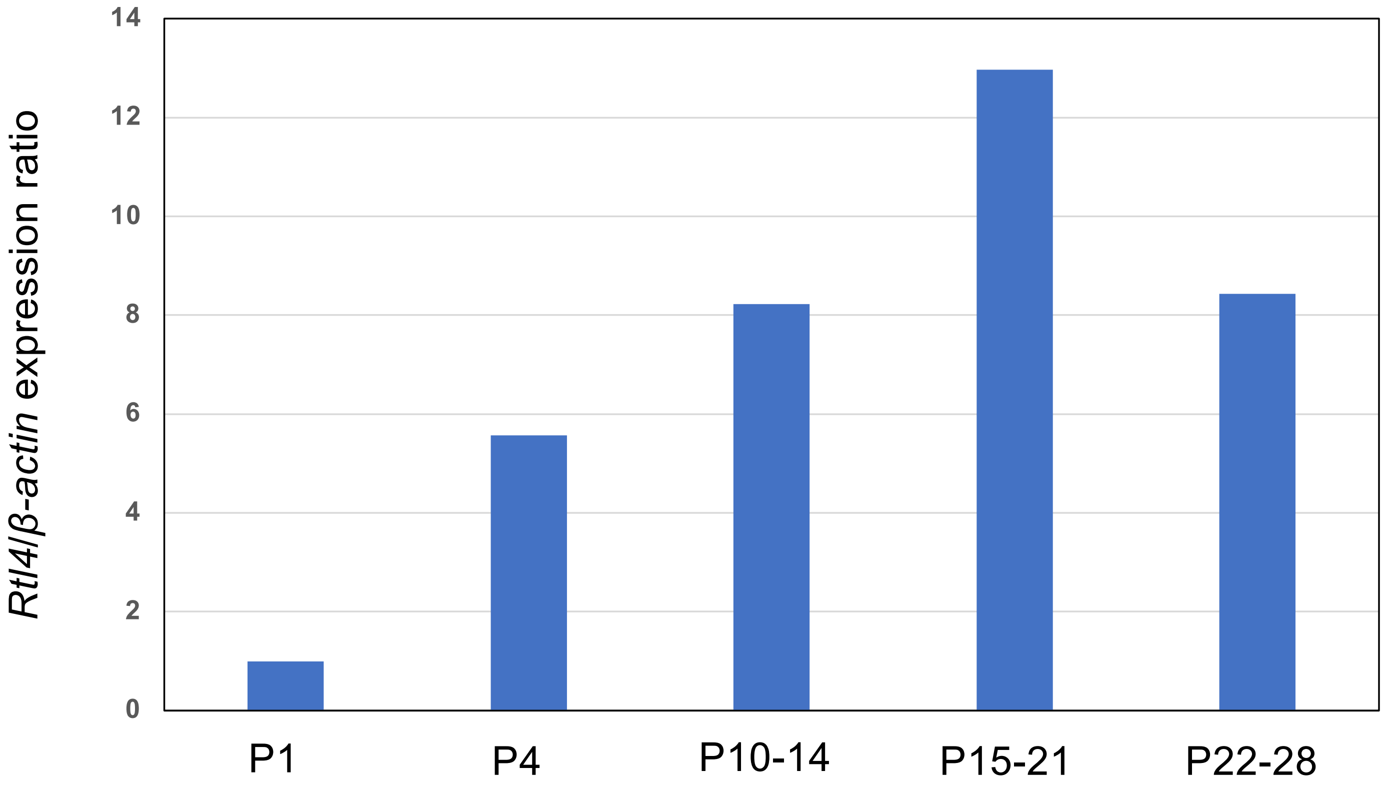

Fig. S2 Postnatal *Rtl4* mRNA expression in WT brain

Both the *Rtl4* and *β-actin* levels were calculated with qPCR, and the ratio of *Rtl4*/*β-actin* expression was adjusted to 1 using the P1 brain level as 1. In the postnatal brain, the expression level of *Rtl4* mRNA increased gradually and peaked at 2-3 weeks. Even at P15-21 (total 14 samples), the threshold cycle (CT) of *Rtl4* was 29.0 ± 0.41 (Mean ± SD), whereas that of *β-actin* was consistently 16.7 ± 0.39 during P1 to P28 (total 52 samples), indicating that the level of *Rtl4* is approximately 1/4,000 of *β-actin*.
PCR primers: *Rtl4*-F5: 5’-AAGAGGAGGATAGGAAATCACTTTG-3’ and *Rtl4*-R5: 5’-GTTGTTAGGACAAGGTTGAGG-3’; *Actb*-F: 5’-AAGTGTGACGTTGACATCCG-3’ and *Actb*-R: 5’-GATCCACATCTGCTGGAAGG-3’.

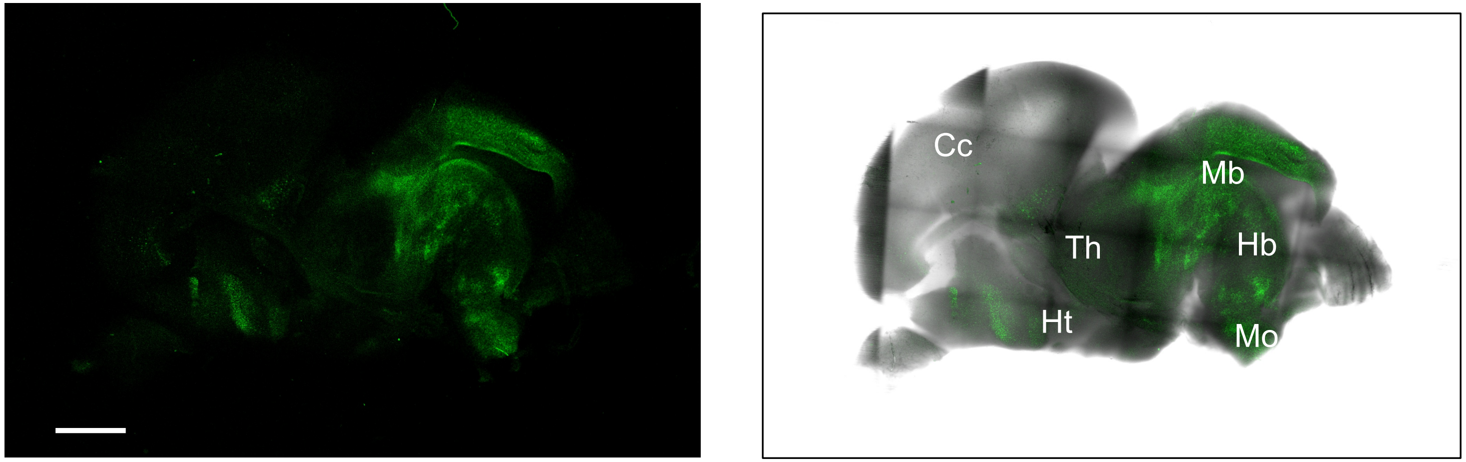

P1 brain: enhanced Venus image

The enhanced Venus image merged with a transmission image

Fig. S3 Expression of RTL4CV protein in P1 brain

RTL4CV expression was detected in certain restricted areas of the P1 brain. Left: The Venus fluorescence image. Right: The fluorescence image merged with a transmission image. Cc: cerebral cortex. Hb: hindbrain. Ht: hypothalamus. Mb: midbrain. Mo: medulla oblongata, Th: thalamus. Bar: 1 mm.

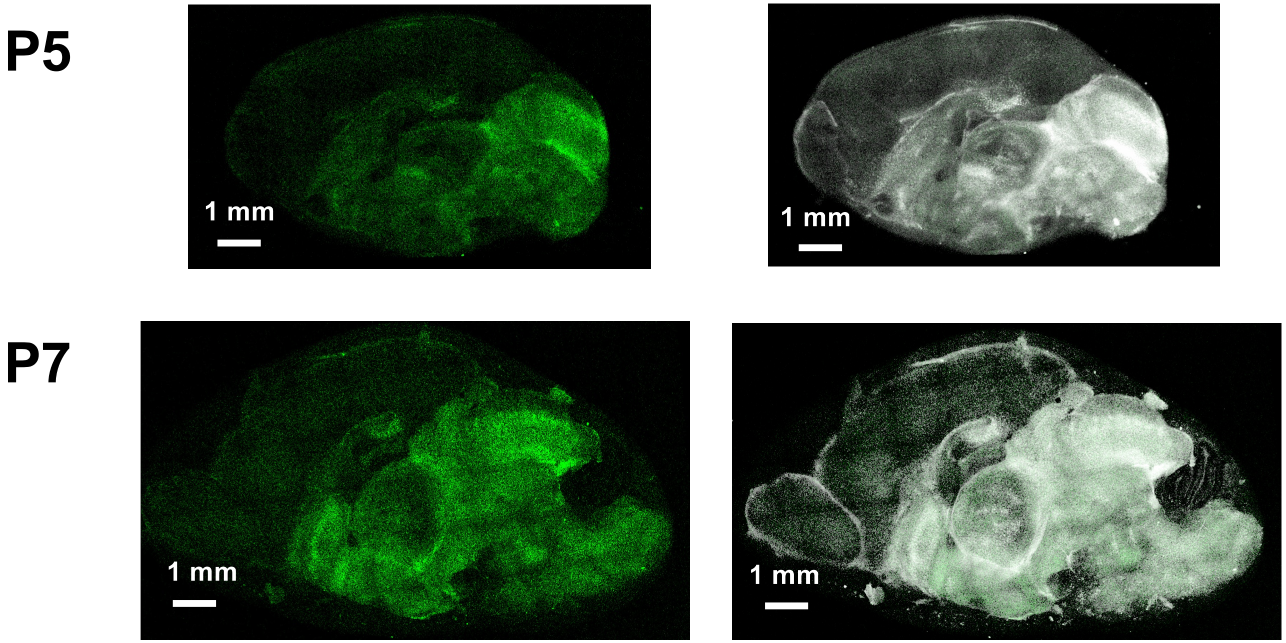

Fig. S4 RTL4CV expression in P5 and P7 brain

Top: In the P5 brain, a strong signal was detected in the midbrain. Middle and bottom: In the P7 brain, the hypothalamus signal became stronger, but the midbrain signal was most significant.

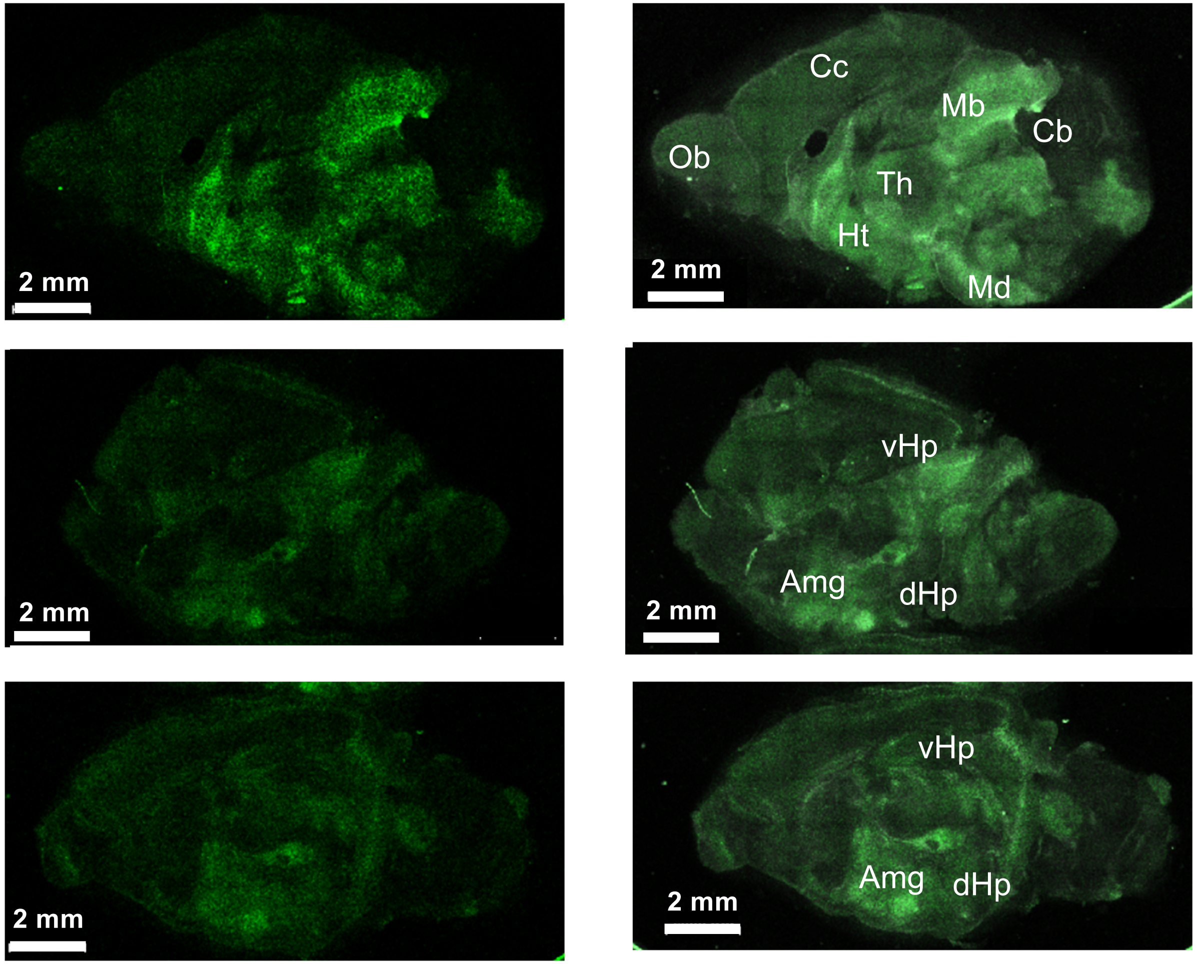

Fig. S5 RTL4CV expression in amygdala in P10 brain

A strong Venus signal was confirmed in the amygdala as well as in the midbrain and hypothalamus in the P10 brain. Venus images of the inner surface of the brain hemisphere (top) and brain slices of 1.5 mm width (middle and bottom). Enhanced Venus images have been presented. Left: Venus images. Right: Venus images merged with autofluorescence images.

P14 cortex

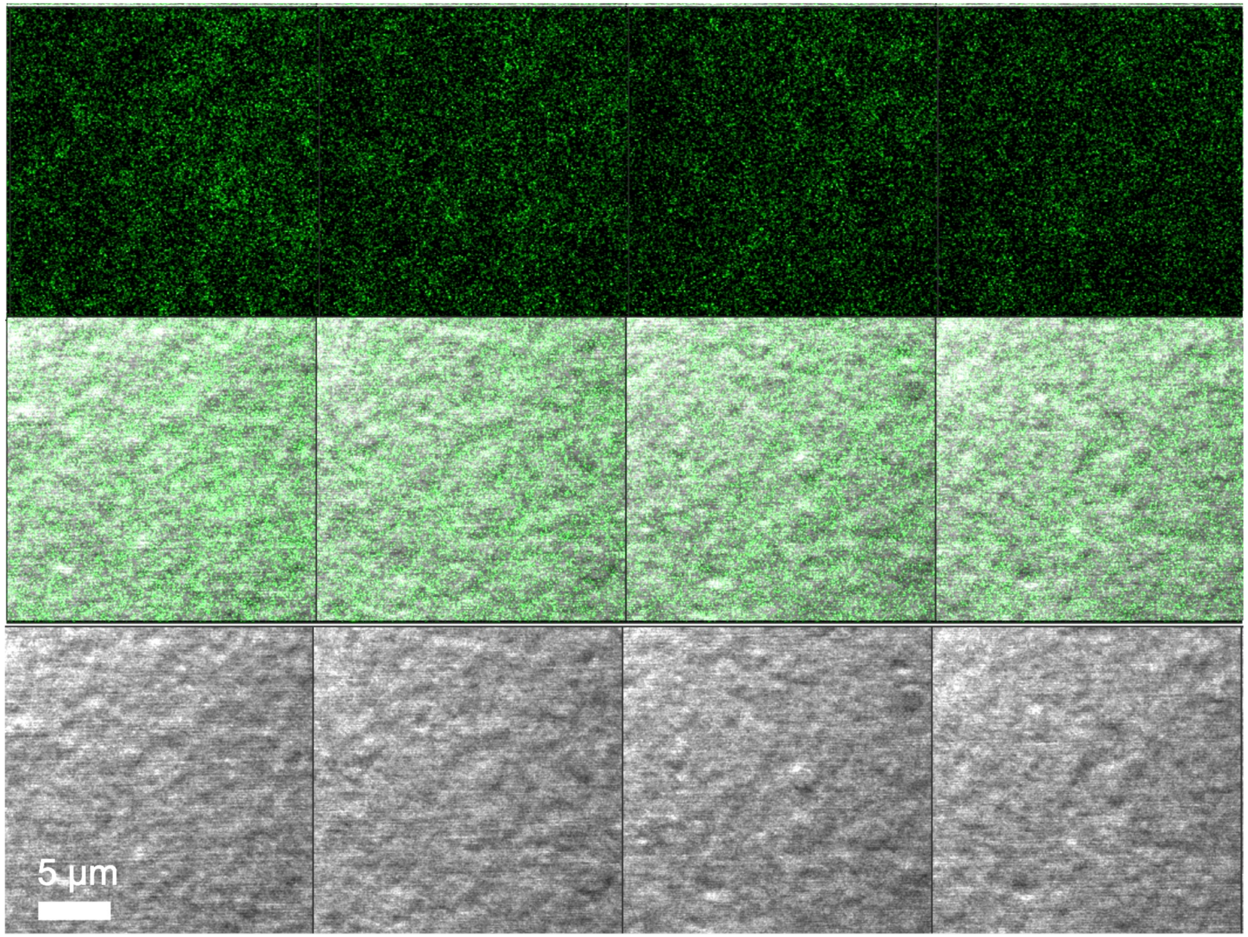

Fig. S6 Widespread dot-like localization of the RTL4CV in the P14 cortex

RTL4CV was detected as widely dispersed dot-like signals, suggesting that it existed as a secretory protein in these brain regions.
P14 cortex Top: : Venus images. Middle: Venus images merged with transmission images. Bottom: Transmission images.
Four continuous 3-D images are displayed at 0.9 µm intervals.

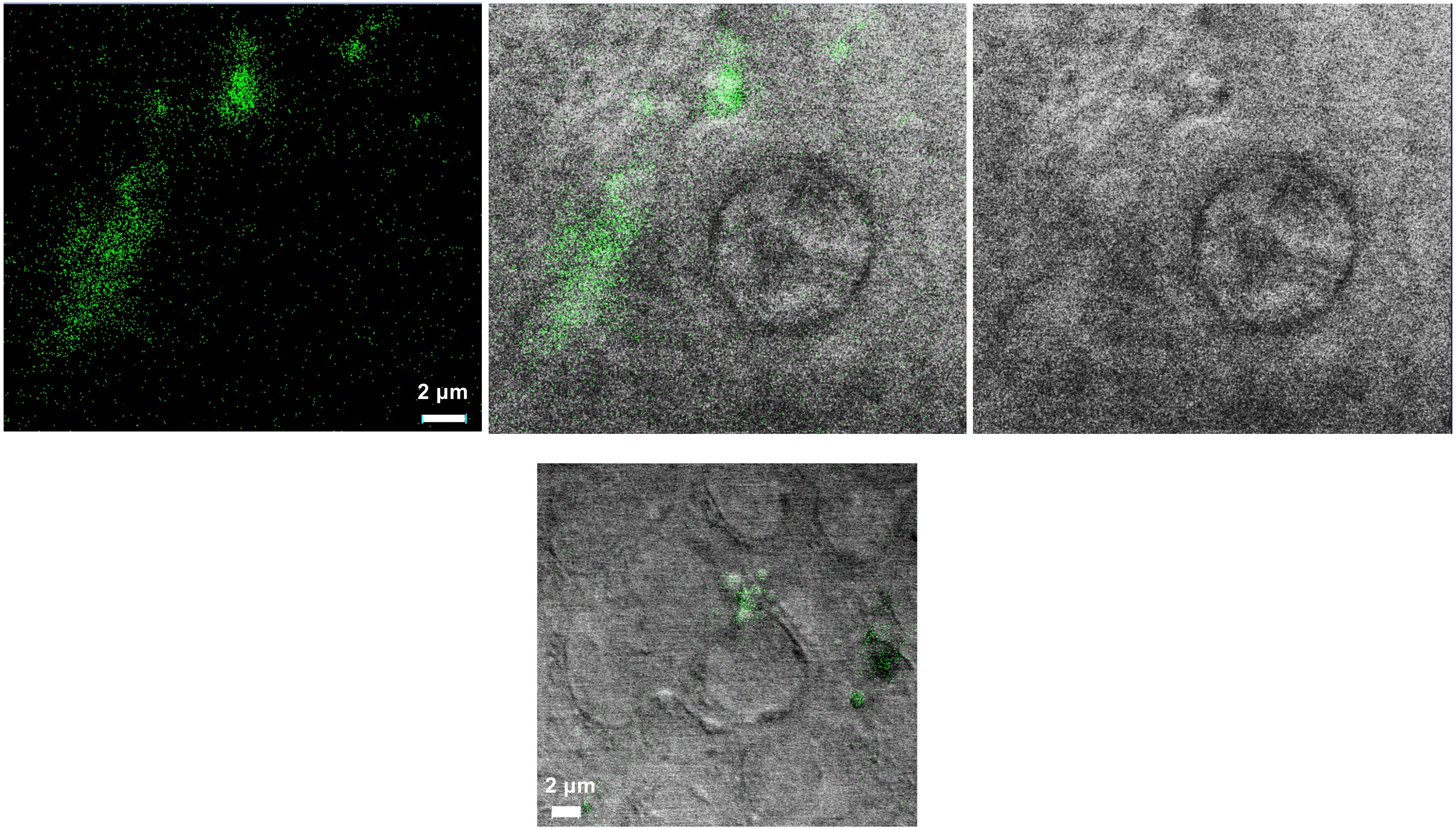

Fig. S7 Presumed extracellular localization of 1 μm granules in the P2 hippocampus

Top: Several 1 μm granules were observed along the cell border. Left: Venus image. Middle: Venus image merged with transmission image. Right: Transmission image. Bottom: Another example (Venus image merged with transmission image).

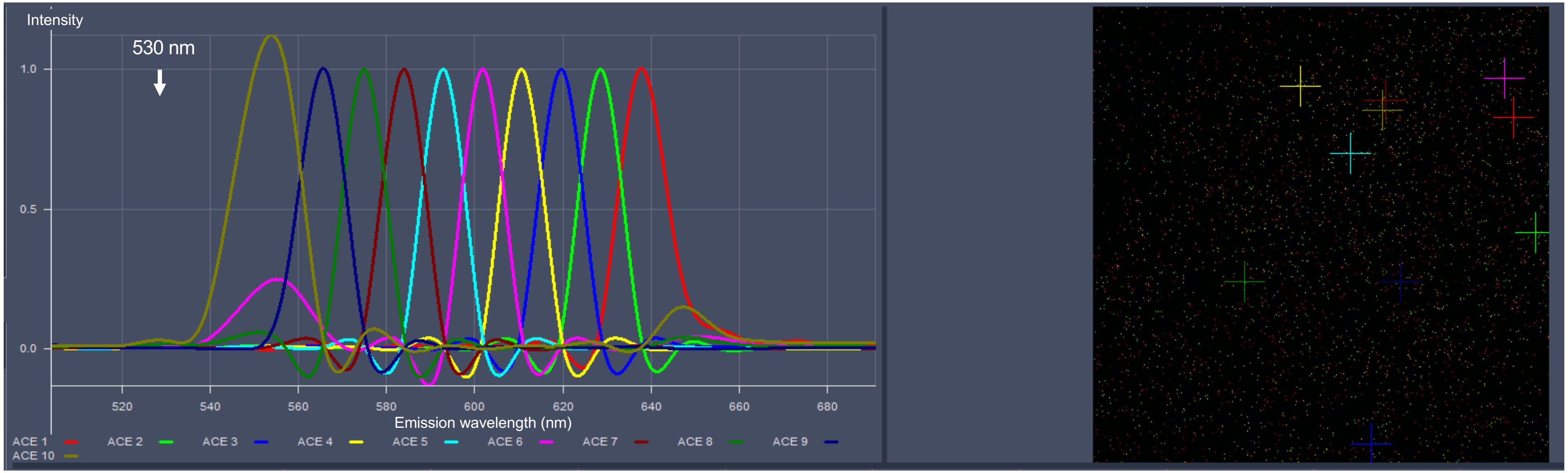

Fig. S8 WT P21 brain analyzed by the Automatic Component Extraction (ACE) function

The hypothalamus of the WT P21 brain was analyzed using the ACE function. There is no waveform with a peak at 530 nm in the WT brain. See also the result of the *Rtl4*CV P15 brain (Fig. 1C). Left: Results from ACE1 to ACE 10. Right: The analyzed hypothalamus region. Each colored cross corresponds to the region from which the corresponding colored waveform (left) was extracted.

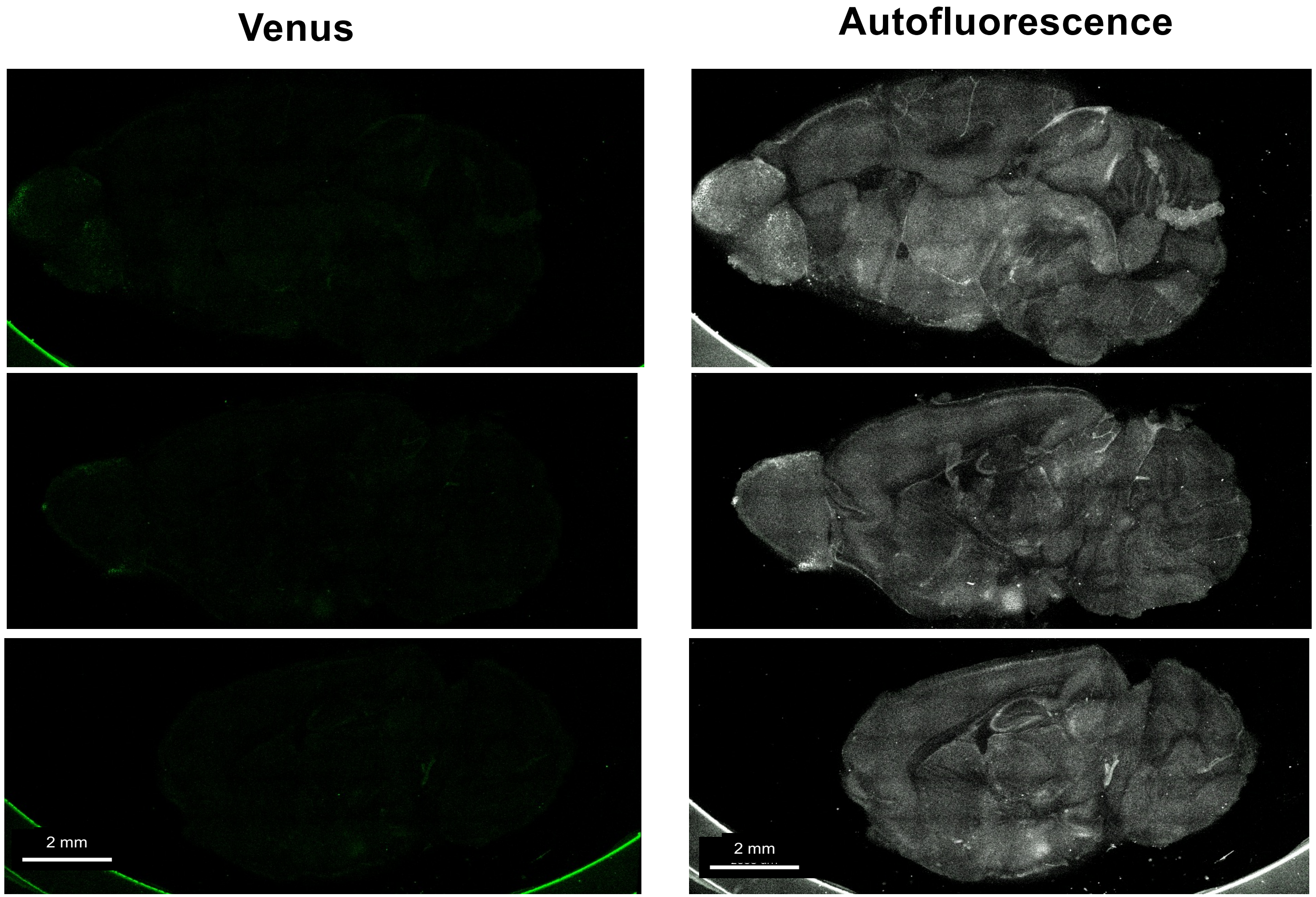

Fig. S9 No Venus signal in WT P21 brain

In the WT brain, the waveform with a peak at 530 nm could not be detected in the top 10 (Fig. S8). The expression pattern in the WT brain was then calculated using the Venus control waveform. The signal intensity was also very low and was treated as background (BG) (see Figs. 2A and B).

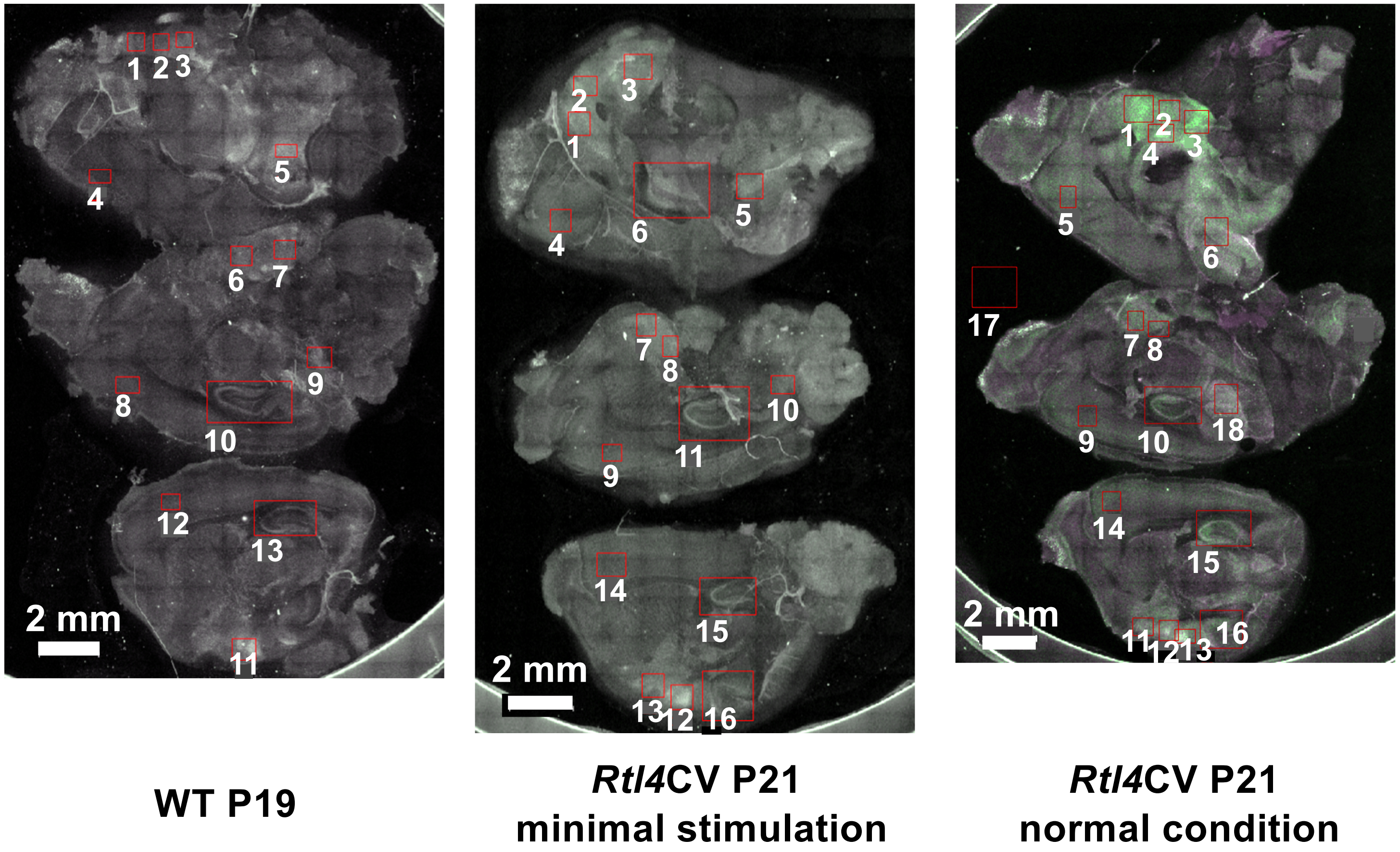

Fig. S10 Environmental effect on the expression of RTL4CV protein in P21 brain

To calculate the Venus signal intensity in each brain region, the boxed areas were measured. The average of three to four parts in the hypothalamus and that of one to four parts in the amygdala are shown in Figure 2B. Top: Inner side of the brain hemisphere. Middle and bottom: Surfaces of brain slices of 1.5 mm width, inner (middle) and outer (bottom) images. Autofluorescence images were merged with Venus images to clarify brain regions. Note that the top two images are upside down: For example, the hypothalamus corresponds to squares 1, 2 and 3 in the left and middle figures, and to squares 1, 2, 3 and 4 in the right figure; the midbrain corresponds to squares 5 and 9 in the left figure, to squares 5 and 10 in the middle figure, and to squares 6 and18 in the right figure; the amygdala corresponds to squares 6, 7 and 11 in the left figure, to squares 7, 8 12 and 13 in the middle figure and to squares 7, 8,11,12 and 13 in the right figure.

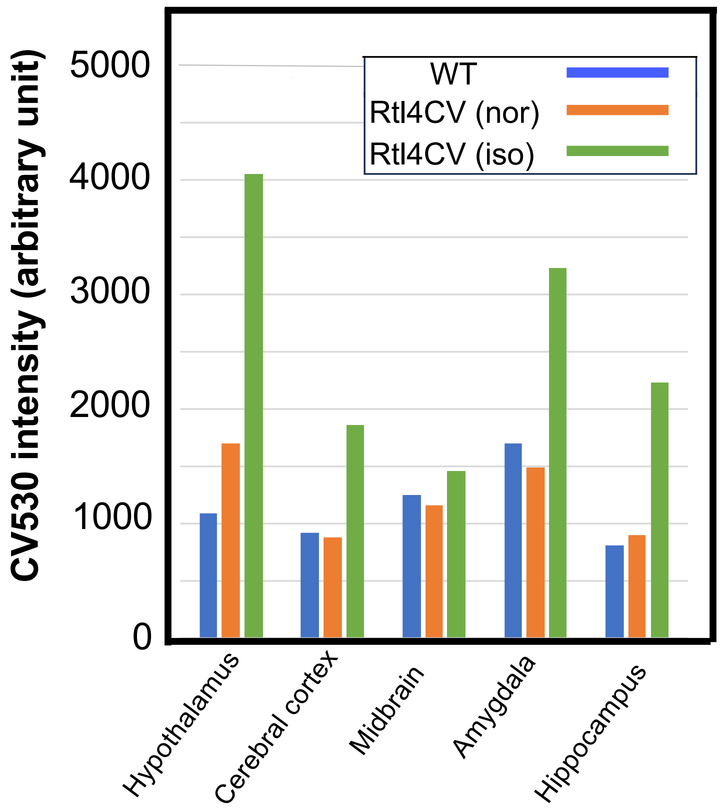

Fig. S11 Another example of isoproterenol administration in P28 brain

Isoproterenol administration experiment in P28 mice: A similar increase in RTL4CV signal was obtained in brain regions at P28 as at P21 (Fig. 2A), but the basal expression levels are different from those at P21, so the data are presented as a separate figure.

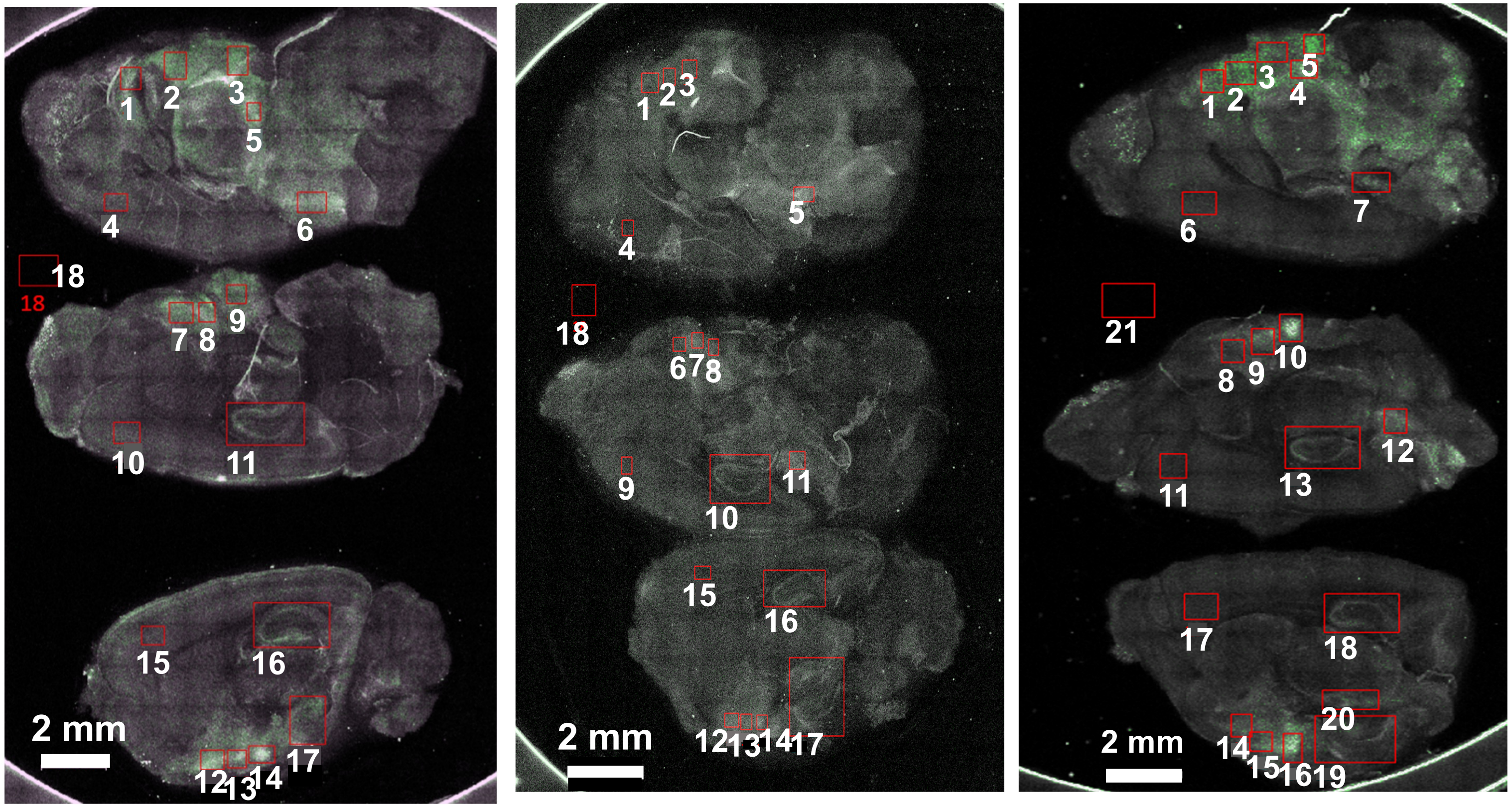

***Rtl4*CV P20**
normal condition

***Rtl4*CV P20**
Saline injection under anesthesia

***Rtl4*CV P20**
Milnacipran injection under anesthesia

Fig. S12 Effect of anesthesia and milnacipran on the expression of RTL4CV protein in P20 brain

Brain regions analyzed on Fig. 2D. Venus images merged with transmission images. See also Fig. S10 legend for analysis of each part of the brain regions. Autofluorescence images were merged with Venus images to clarify brain regions.

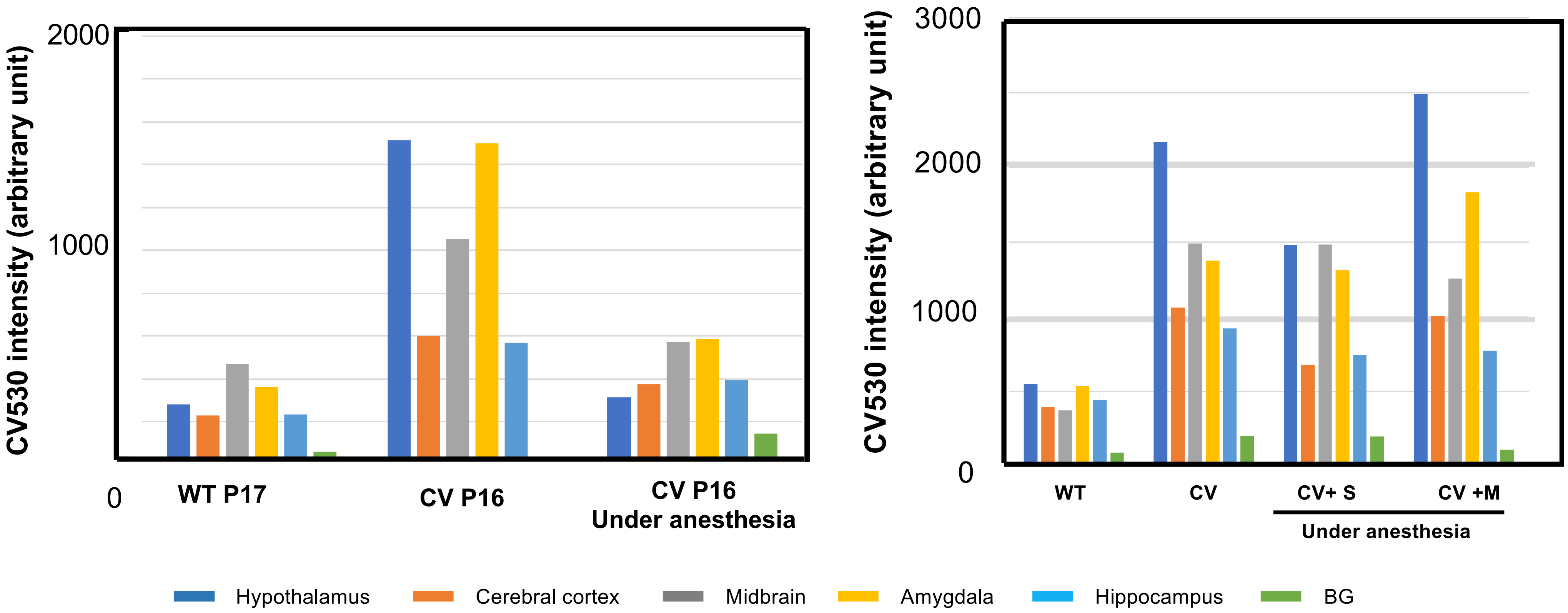

Fig. S13 Another example of isoflurane anesthesia and milnacipran administration

Left: Isoflurane anesthesia in P16 brain. A similar decrease in RTL4CV signal under anesthesia was obtained in brain regions at P16 as at P27 (Fig. 2D), but the basal expression levels are different from those at P27, so the data are presented as a separate figure. Right: Another example of milnacipran administration in P27 brain.

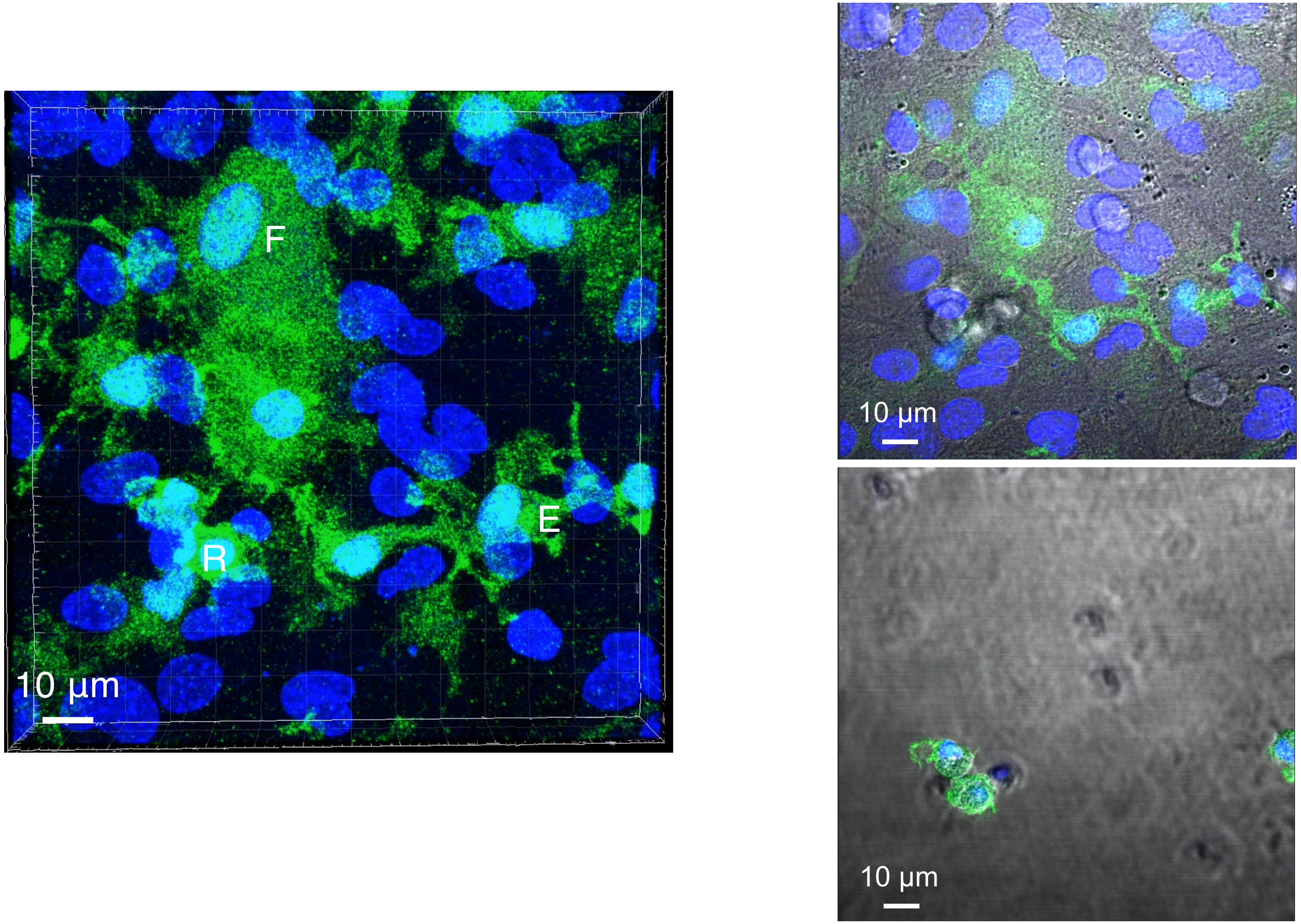

Fig. S14 Microglial cells in the primary mixed glial culture (Iba1 staining)

Several types of microglia were present in the primary mixed glial culture. Top left: flat cells below and/or within, and those with multiple processes on the astrocyte feeder layer. Bottom left: round floating cells above the feeder layer. Right: 3D movie of the primary mixed glial culture. Immunostaining with anti-Iba1-Alexa488 antibody (artificial green. DAPI (blue). Bar: 10 µm. See also 3D movie of Iba1 staining of mixed glia culture.

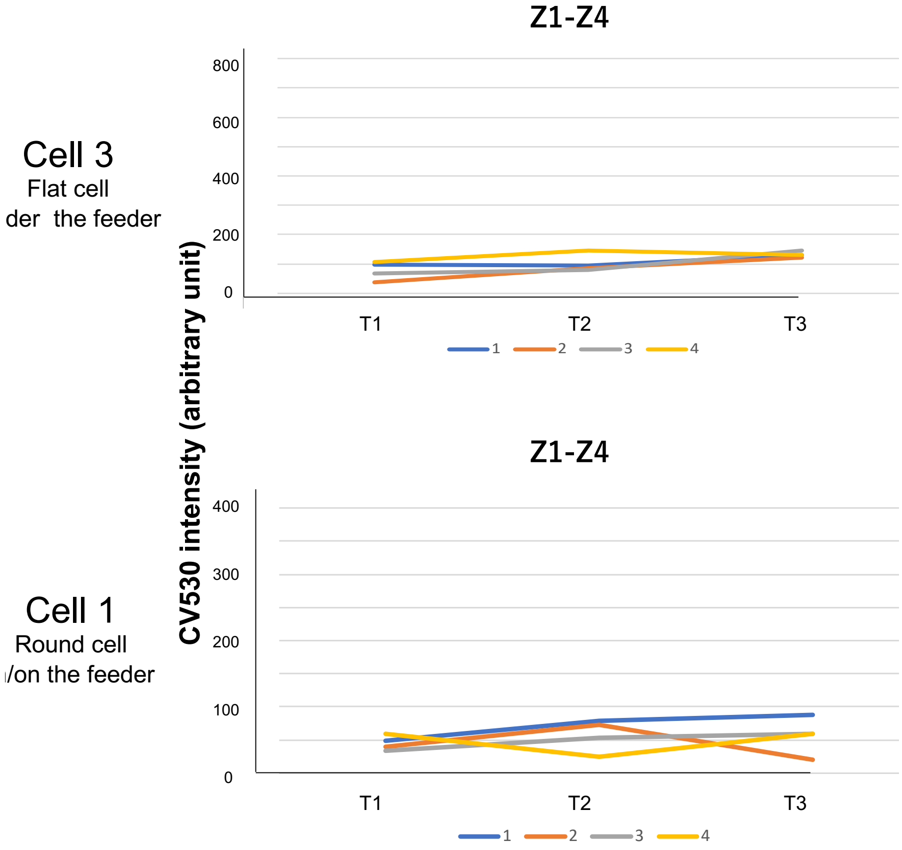

Fig. S15-1 Time-lapse experiment using mixed glial culture (isoproterenol administration)
The signal intensity of Z1-4 (near glass bottom) of Cell 3 (Top) and Cell 1 (Bottom) of Fig. 3C.

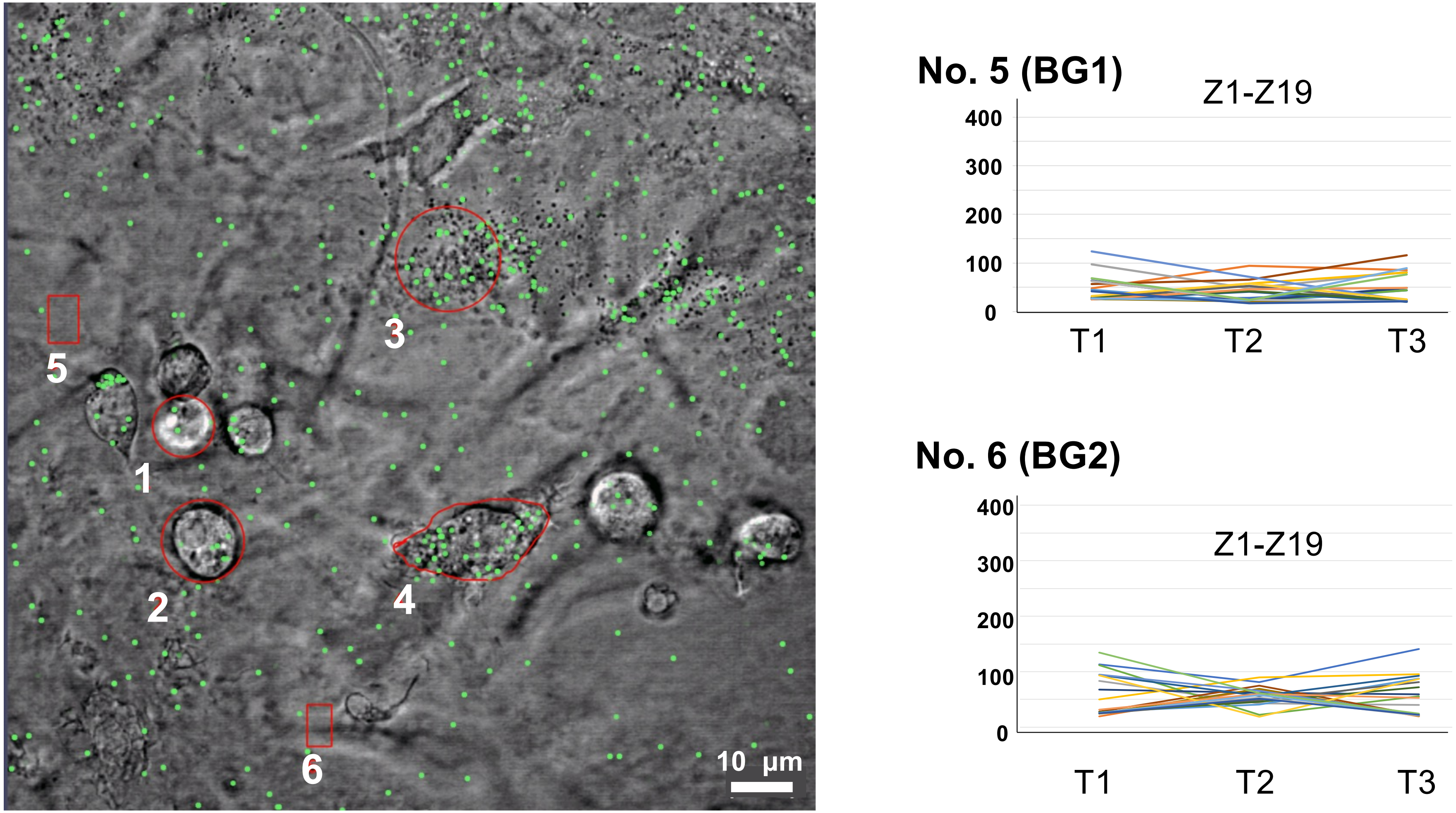

Fig. S15-2 Time-lapse experiment using mixed glial culture (isoproterenol administration)

Left: photo of the mixed glial culture analyzed in Fig. 3C. The areas surrounded by red lines were measured: the circles (1-3) and the outlined area (4) correspond to microglial cells and the boxes (5 and 6, astrocyte feeder layer only) correspond to BG. Right: the results of the time-lapse experiment of two BG regions. The results of Cells 1 and 3 are presented in Fig. 3C and Fig. S15-1.

Table S1 Primers used for *Rtl4*CV mouse generation

Experiment	Primer name	Sequence (5’ to 3’)
Single-stranded DNA production	PCR-F	TGCGTCCACTACCAAAGGAT
	PCR-R	GAGGAGGGGCTATCTTTCAAAC
Genotyping	Primer 1	CCAGATTTGATCACTCAGTGC
	Primer 2	TGTTGTGGCGGATCTTGAAG
	Primer 3	AGCAGCACGACTTCTTCAAG
	Primer 4	CTCTTGAAGCTGATTGGTCC
